# Stepwise diagnostic algorithm for high-attenuation pulmonary abnormalities on CT

**DOI:** 10.1186/s13244-023-01501-x

**Published:** 2023-10-20

**Authors:** Taiki Fukuda, Ryoko Egashira, Midori Ueno, Mikiko Hashisako, Hiromitsu Sumikawa, Junya Tominaga, Daisuke Yamada, Junya Fukuoka, Shigeki Misumi, Hiroya Ojiri, Hiroto Hatabu, Takeshi Johkoh

**Affiliations:** 1 Department of Radiology, The Jikei University School of Medicine, 3-25-8, Nishi-Shimbashi, Minato-Ku, Tokyo, 105-8461 Japan; 2 Department of Radiology, Faculty of Medicine, Saga University, 5-1-1, Nabeshima, Saga-City, Saga 849-8501 Japan; 3 Department of Radiology, University of Occupational and Environmental Health, 1-1, Iseigaoka, Yahatanishi-Ku, Kitakyushu, Fukuoka 807-8556 Japan; 4 Department of Pathology, Kyushu University, 3-1-1, Maidashi, Higashi-Ku, Fukuoka-City, Fukuoka 812-8582 Japan; 5 Department of Radiology, National Hospital Organization Kinki-Chuo Chest Medical Center, 1180, Nagasone-Cho, Kita-Ku, Sakai-City, Osaka 591-8555 Japan; 6 Department of Diagnostic Radiology, Tohoku University Graduate School of Medicine, 1-1, Seiryo-Machi, Aoba-Ku, Sendai, 980-8574 Japan; 7 Department of Radiology, St. Luke’s International Hospital, 9-1, Akashicho, Chuo-Ku, Tokyo, 104-8560 Japan; 8 Department of Pathology, Nagasaki University Graduate School of Biomedical Sciences, 1-7-1, Sakamoto, Nagasaki-City, Nagasaki 852-8523 Japan; 9 Department of Radiology, Brigham and Women’s Hospital, Harvard Medical School, 75 Francis Street, Boston, MA 02115 USA; 10 Department of Radiology, Kansai Rosai Hospital, 3-1-69, Inabaso, Amagasaki, Hyogo 660-8511 Japan

**Keywords:** Lung diseases, Tomography (X-ray computed), Algorithms, High attenuation, Calcification

## Abstract

High-attenuation pulmonary abnormalities are commonly seen on CT. These findings are increasingly encountered with the growing number of CT examinations and the wide availability of thin-slice images. The abnormalities include benign lesions, such as infectious granulomatous diseases and metabolic diseases, and malignant tumors, such as lung cancers and metastatic tumors. Due to the wide spectrum of diseases, the proper diagnosis of high-attenuation abnormalities can be challenging. The assessment of these abnormal findings requires scrutiny, and the treatment is imperative. Our proposed stepwise diagnostic algorithm consists of five steps. Step 1: Establish the presence or absence of metallic artifacts. Step 2: Identify associated nodular or mass-like soft tissue components. Step 3: Establish the presence of solitary or multiple lesions if identified in Step 2. Step 4: Ascertain the predominant distribution in the upper or lower lungs if not identified in Step 2. Step 5: Identify the morphological pattern, such as linear, consolidation, nodular, or micronodular if not identified in Step 4. These five steps to diagnosing high-attenuation abnormalities subdivide the lesions into nine categories. This stepwise radiologic diagnostic approach could help to narrow the differential diagnosis for various pulmonary high-attenuation abnormalities and to achieve a precise diagnosis.

**Critical relevance statement** Our proposed stepwise diagnostic algorithm for high-attenuation pulmonary abnormalities may help to recognize a variety of those high-attenuation findings, to determine whether the associated diseases require further investigation, and to guide appropriate patient management.

**Key points**

• To provide a stepwise diagnostic approach to high-attenuation pulmonary abnormalities.

• To familiarize radiologists with the varying cause of high-attenuation pulmonary abnormalities.

• To recognize which high-attenuation abnormalities require scrutiny and prompt treatment.

**Graphical Abstract:**

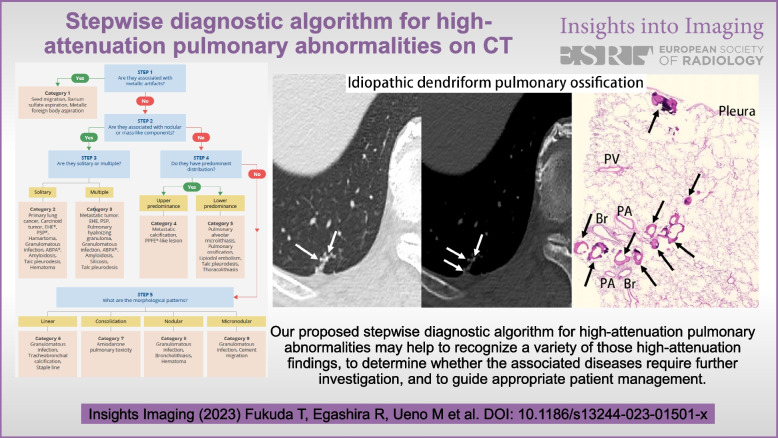

**Supplementary Information:**

The online version contains supplementary material available at 10.1186/s13244-023-01501-x.

## Background

High-attenuation pulmonary abnormalities on CT are often caused by calcification that develops during the healing process of infectious granulomatous diseases. Thus, such abnormalities are usually considered to be non-significant findings. However, they may also be encountered in tumors, metabolic disorders, and occupational diseases. Furthermore, substances other than calcification with high atomic numbers, such as manganese, iron, platinum, barium, iodine, and mercury, also cause high-attenuation pulmonary abnormalities. With the widespread use of thin-slice CT, the frequency of detecting tiny high-attenuation lesions, such as those represented by diffuse pulmonary ossification, and the sensitivity of detecting calcification in lung cancers or metastatic tumors is increasing. Therefore, a wide variety of high-attenuation pulmonary abnormalities on CT may be encountered in daily clinical practice, and identifying the type of lesion can reduce the patients’ burden and healthcare costs related to unnecessary scrutiny and surgery. Further, proper identification allows the clinician to promptly determine whether further examination or the initiation of treatment is needed.

This article aims to outline a stepwise diagnostic approach to high-attenuation pulmonary lesions that can be applied in clinical practice and provide insights into their pathological background. We have defined high-attenuation lesions in the lung in this article as areas that exhibit higher attenuation than the surrounding muscle tissue. Thus, they range from lesions that show faint high attenuation compared to skeletal muscle, such as hematomas, to those that show dense high attenuation, such as barium sulfate. This definition encompasses lesions located in both the lung fields and in the bronchial and pleural cavities, which can sometimes be challenging to differentiate from high-attenuation lesions in the lungs.

## Stepwise approach to high-attenuation pulmonary abnormalities

Our proposed stepwise approach consists of five steps in which high-attenuation lesions are classified into nine categories based on the presence or absence of metallic artifacts, morphology, distribution, and concomitant findings. Diseases and findings showing high-attenuation pulmonary abnormalities occupy a broad spectrum of pathologies for which differential diagnosis can be complicated. Take, for example, the differentiation of small granulomatous lesions with calcification from pulmonary ossification. Without knowledge of pulmonary ossification, small high-attenuation lesions in the lower lobes might also be considered granulomatous lesions with calcification. Both are high-attenuation lesions showing linear or micronodular patterns, though pulmonary ossification is primarily predominant in the lower lobes. Therefore, the distinction between both can be made in STEP 4, which highlights the distributional predominance. Following our proposed algorithm, a differential diagnosis can be made even in similar high-attenuation lesions, which can lead to the correct diagnosis.

**Fig. 1:**
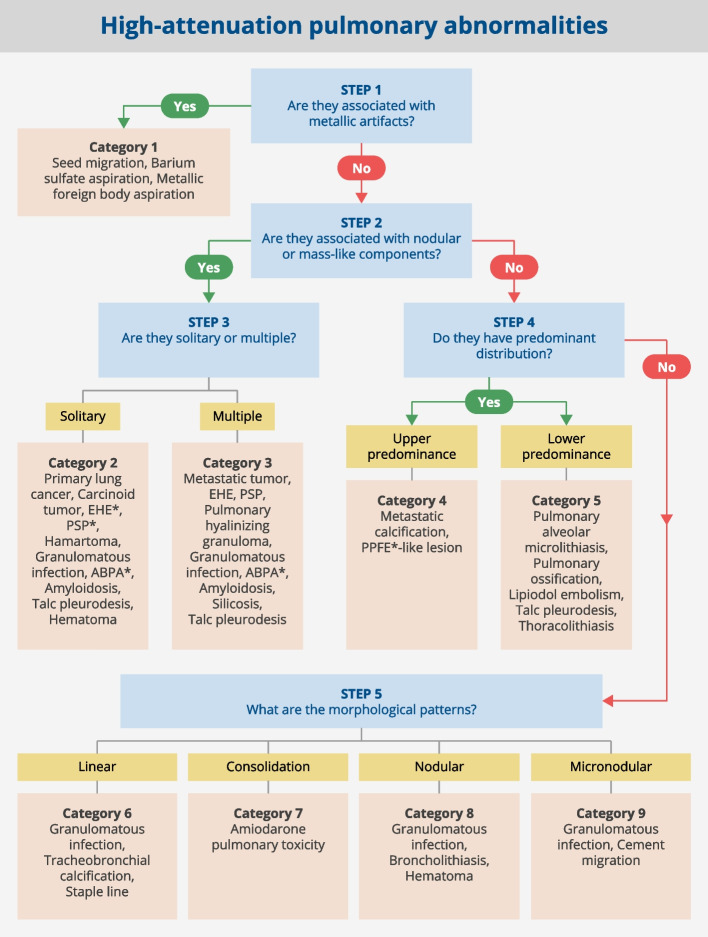
Stepwise diagnostic algorithm for high-attenuation pulmonary abnormalities on CT. Steps 1 to 5 in the flow chart for determining high-attenuation pulmonary abnormalities and the 9 categories of lesions are illustrated. ABPA, allergic bronchopulmonary aspergillosis; EHE, epithelioid hemangioendothelioma; PPFE, pleuroparenchymal fibroelastosis; PSP, pulmonary sclerosing pneumocytoma

A summary of the stepwise approach to high-attenuation pulmonary abnormalities is given in Fig. 1. Although some diseases may fall into more than one category, we describe the diseases within the categories in which such lesions are most frequently encountered.

Thin sections are needed for the imaging evaluation of high-attenuation pulmonary abnormalities on CT. For example, pulmonary ossification is a tiny high-attenuation abnormality; thus, precise assessment requires a gapless scan of 1-mm slice thickness. Furthermore, the optimal assessment of calcification also requires thin sections to minimize volume averaging. Mediastinal window images (e.g., width, 350 HU (Hounsfield unit); level, 60 HU) are suitable for observation to assess mild calcification, whereas bone window (e.g., width, 2500 HU; level, 500 HU) images are suitable for assessing dense calcification, ossification, and metallic substances. Coronal reconstruction is also useful in assessing diseases with a predilection of distribution.

### Step 1: Establish the presence or absence of metallic artifacts

Evaluation of high-attenuation pulmonary abnormalities begins with ascertaining whether the lesions on CT scans are associated with metallic artifacts.

#### Category 1: With metallic artifact

This category comprises abnormalities of non-biological origin, including seed migration, barium sulfate aspiration, and metallic foreign body aspiration.

##### Seed migration

Low-dose brachytherapy with a permanent implant of I-125 seeds is considered standard therapy for patients with localized prostate adenocarcinoma. Migration of I-125 seeds is reported in approximately 25% of patients and occurs in about 0.5% of cases per I-125 seed [1]. After migrating into the venous plexus around the prostate, I-125 seeds can reach peripheral vessels of various organs due to the contraction of the adjacent bladder [2]. Specifically, they may migrate to the iliac veins, right heart, and lungs [3]. Approximately 80% of migrating I-125 seeds are found in the lungs [1] and are characterized by metallic artifacts (Supplementary Fig. S1). The risk of seed migration was lowest when implants were performed exclusively with stranded seeds compared to those using loose seeds [4].

##### Barium sulfate aspiration

Discrete high-attenuation lesions are often caused by aspiration of barium sulfate during an upper gastrointestinal study. The involved lung regions depend on the patient’s position during and after aspiration. Thus, the basal segments of the lower lobes are most often involved (Supplementary Fig. S2). After aspiration, most of the barium particles accumulated in alveolar spaces, specifically those with centrilobular distribution, are phagocytosed by alveolar macrophages. However, others may pass directly across the alveolar epithelium into the alveolar or peribronchiolar interstitial tissue. Some particles are transported via lymphatics in the interlobular septa and to the pleura, whereas others remain in the interstitial tissue. Fibrosis may also occur in areas with residual barium [5]. Although barium is generally non-irritating, simultaneous aspiration of gastric acid, anaphylactic reactions, and massive aspiration can lead to severe hypoxia, acute respiratory distress syndrome (ARDS), and even death [6, 7].

##### Metallic foreign body aspiration

Representative inhaled metallic foreign bodies are pins, screws, and dental prostheses [8, 9]. In adults, due to the vertical orientation of the right main bronchus, the most common location for a foreign body in the airway is the right bronchial tree, particularly in the right lower lobe and bronchus intermedius (Supplementary Fig. S3) [8].

### Step 2: Identify associated nodular or mass-like soft tissue components

For lesions without associated metallic artifacts, perform Step 2 to identify whether the high-attenuation lesions are associated with nodular or mass-like soft tissue-attenuation components. If so, skip to Step 3; otherwise, skip to Step 4.

### Step 3: Establish the presence of solitary or multiple lesions

This step is performed to identify whether solitary or multiple nodular or mass-like components accompanied by high-attenuation lesions are present.

#### Category 2: Solitary nodular or mass-like component

Tumors and granulomas are often present in the background.

##### Primary lung cancer

**Fig. 2:**
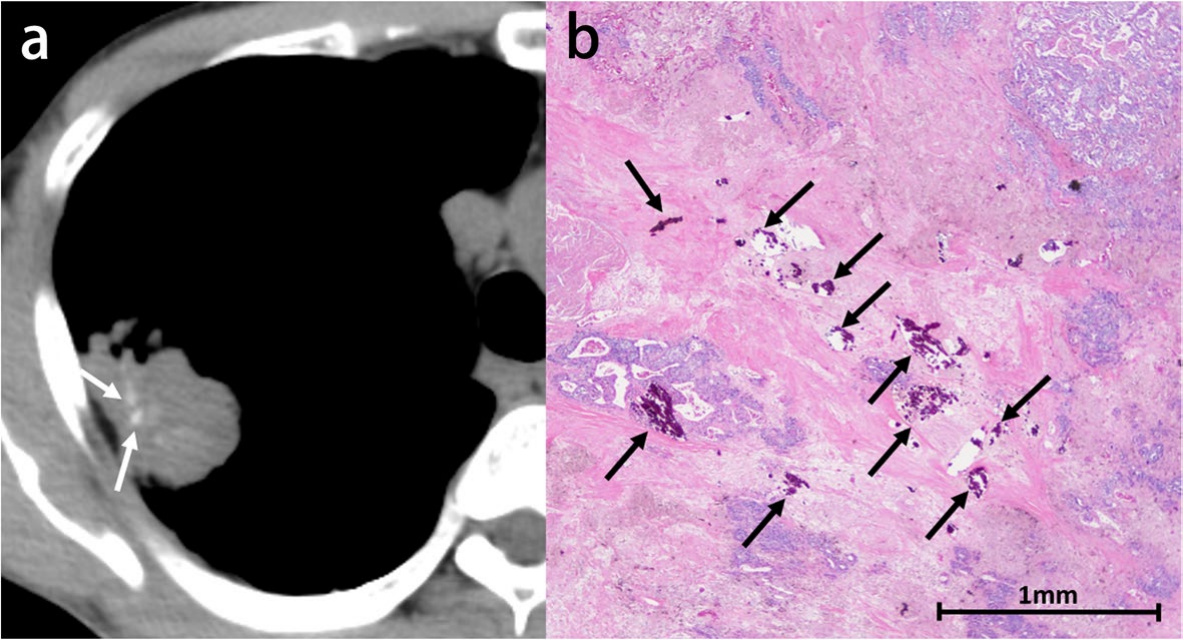
Invasive adenocarcinoma in a 70-year-old man. **a** CT image shows a right upper lobe mass with eccentric punctate and amorphous calcification (arrows). **b** Photomicrograph (H-E stain) shows invasive adenocarcinoma with extensive necrosis and calcification (arrows)

**Fig. 3:**
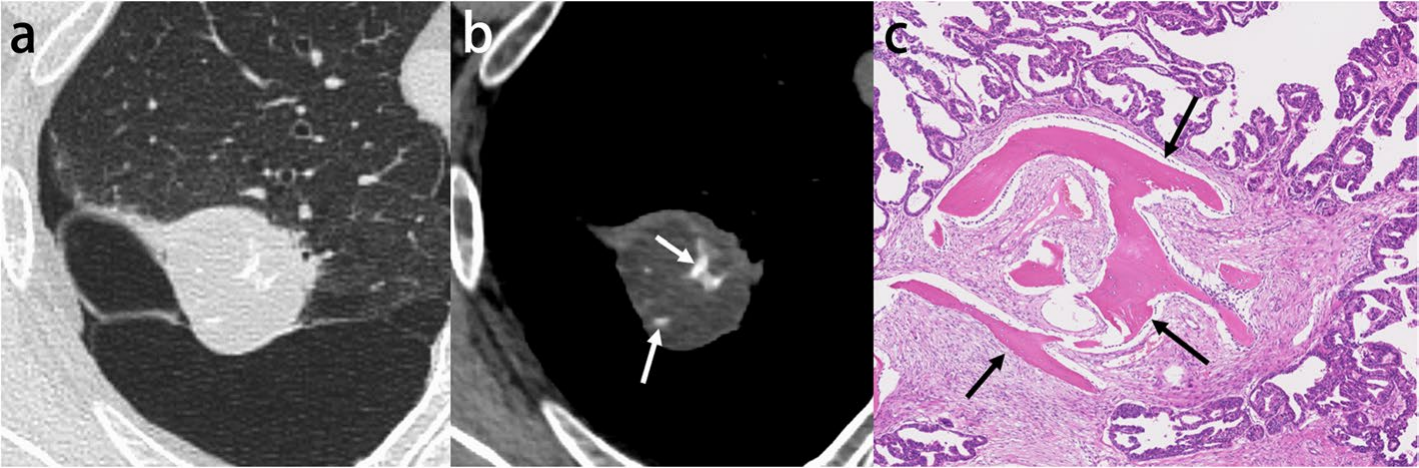
Invasive adenocarcinoma with stromal ossification in a 69-year-old man. **a** CT image shows a right upper lobe mass adjacent to the emphysematous cysts. **b** Mediastinal window image reveals punctate and amorphous calcification or ossification in the mass (arrows). **c** Photomicrograph (original magnification, × 5.6; H-E stain) shows invasive adenocarcinoma with stromal ossification (arrows)

Calcification is identified in 6–10% of lung cancers, although it does not predict histologic subtype and tends to occur in large tumors [10, 11]. Several mechanisms of calcification in lung cancers have been proposed: engulfment of calcific scar tissue or granulomatous disease, degenerated bronchial cartilage, dystrophic calcification in areas of tumor necrosis, and the secretory function of the carcinoma itself. Calcification may also develop as a sequela of chemotherapy [10, 12]. Furthermore, immune checkpoint inhibitor therapy may cause calcification within the tumor due to drug-induced tumor necrosis [13]. Calcification occurs with amorphous, punctate, or reticular patterns (Fig. 2). Although rare, heterotopic ossification within lung carcinoma may also be present (Fig. 3) [14, 15].

##### Carcinoid tumor

Bronchial carcinoids are neuroendocrine tumors that range from low-grade typical carcinoids to more aggressive atypical carcinoids. They comprise only 1–2% of all lung tumors and arise in the bronchial and bronchiolar epithelium. Carcinoids may calcify diffusely, and eccentric calcifications are common with foci of calcification or even ossification seen at histologic analysis in up to 30% of cases, particularly in central carcinoids [16].

##### Pulmonary sclerosing pneumocytoma

Pulmonary sclerosing pneumocytoma (PSP), formerly known as pulmonary sclerosing hemangioma, is a relatively rare, slow-growing, benign tumor that develops in middle-aged women. Although PSP generally presents as a solitary well-defined nodule, multiple nodules may also occur [17]. PSP appears as a well-defined mass with good contrast enhancement ranging from 96 to 157 HU. In addition, the lesion may harbor high-, iso-, and low-attenuation areas that correspond to the tumor’s angiomatous, solid/sclerotic, and cystic components [18].

**Fig. 4:**
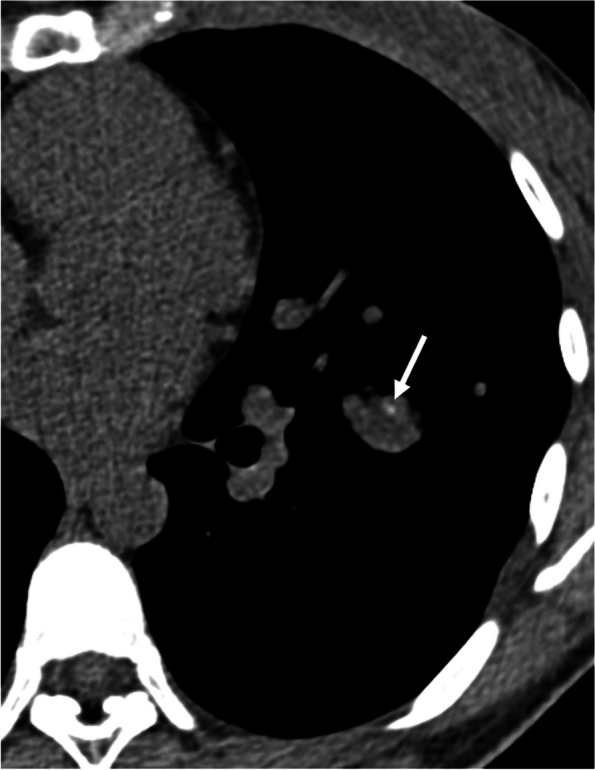
Pulmonary sclerosing pneumocytoma in a 25-year-old woman. CT image shows punctate calcifications within the large nodule (arrow)

Calcification has been reported in 41% of PSP on microscopic examination [19]. However, the frequency of calcification on CT is less frequent than that in pathology, with seven reports of calcification among 46 cases [20]. Furthermore, Chung et al. reported that 30% of PSP nodules on CT contained an intratumoral calcification, all of which were stippled (Fig. 4) [21].

##### Hamartoma

Pulmonary hamartoma is the most common mesenchymal tumor of the respiratory system, accounting for more than 75% of all benign lung tumors. Furthermore, hamartoma comprises varying amounts of at least two mesenchymal elements, including cartilage, fat, connective tissue, and smooth muscle, combined with respiratory epithelium.

On high-resolution CT (HRCT), fat is identified in 34–50% of pulmonary hamartoma and calcification in 15–30%. The frequency of calcification increases with tumor size [12]. Calcification of pulmonary hamartomas, which results from the calcification of cartilage, can be punctate or conglomerate (Supplementary Fig. S4) [22]. Popcorn calcification is classically described as a CT finding of hamartoma, although it is seen infrequently [23].

#### Category 3: Multiple nodular or mass-like components

Granulomatous diseases are the most prevalent. However, tumors, such as multiple lung metastases and epithelioid hemangioendothelioma (EHE), may also develop. Amyloidosis is also an important differential diagnosis.

##### Metastatic tumor

**Fig. 5:**
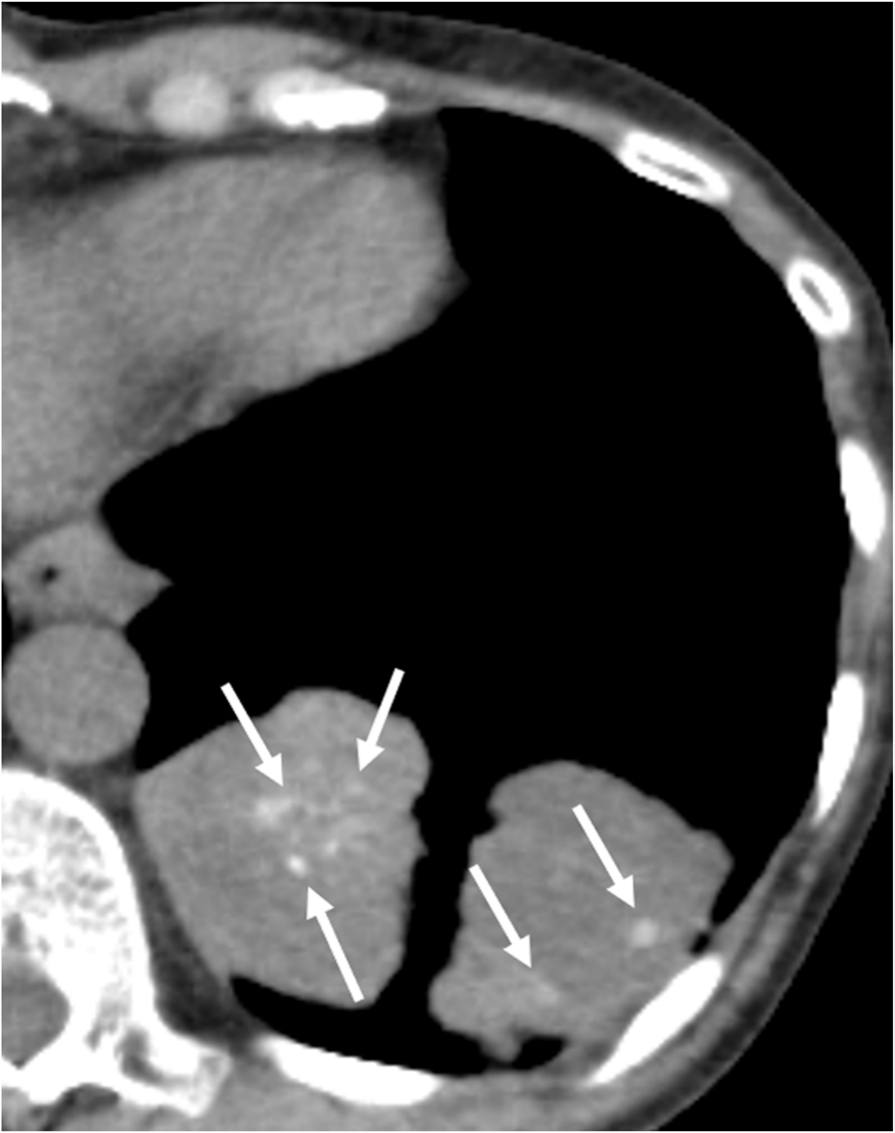
Metastatic colorectal cancer in a 61-year-old woman. CT image shows soft tissue masses with multiple punctate calcifications in the left lower lobe (arrows)

Calcification or ossification commonly occurs with metastatic osteosarcoma and chondrosarcoma. In addition, metastatic carcinomas of the colon (Fig. 5), ovary, breast, and thyroid (Supplementary Fig. S5), and metastatic synovial sarcoma and giant cell tumors of the bone, may calcify. Several mechanisms are responsible for calcification: first, bone formation in osteosarcoma or chondrosarcoma; second, dystrophic calcification in papillary carcinoma of the thyroid, giant cell tumor of the bone, synovial sarcoma, or treated metastatic tumor; and third, mucoid calcification in mucinous adenocarcinoma of the gastrointestinal tract and breast [24]. Calcification may persist following successful chemotherapy despite resolution of the tumor [13].

##### Epithelioid hemangioendothelioma

EHE is a rare tumor of vascular origin, which presents an intermediate clinical course between hemangioma and angiosarcoma. Multiple small nodular pattern (< 15 mm in diameter) and multiple large nodular pattern (≥ 15 mm in diameter) were observed in 71.4% and 22.9% of patients, respectively. However, 5.7% of EHE showed a solitary lesion and may mimic lung cancer [25]. EHE was located predominantly subpleurally with a perivascular location, and 48% of the nodules/masses showed punctate calcification [26]. Extra-pulmonary EHE lesion were observed in 62.9% of cases, with most lesions occurring in the liver. Even with multiple nodules, the patient is expected to have a good prognosis and may not need urgent treatment. Furthermore, spontaneous regression may also occur [25].

##### Pulmonary hyalinizing granuloma

Pulmonary hyalinizing granuloma (PHG) is a rare benign lung disease that presents as single or multiple lung nodules. The exact etiology is still unknown, although it is hypothesized to be an exaggerated immune response to antigenic stimuli caused by infection or an autoimmune process [27]. PHG is also related to other fibro-sclerosing conditions such as retroperitoneal fibrosis, sclerosing mediastinitis, and sclerosing cholangitis, which are part of the spectrum of IgG4-related sclerosing disease [28].

**Fig. 6:**
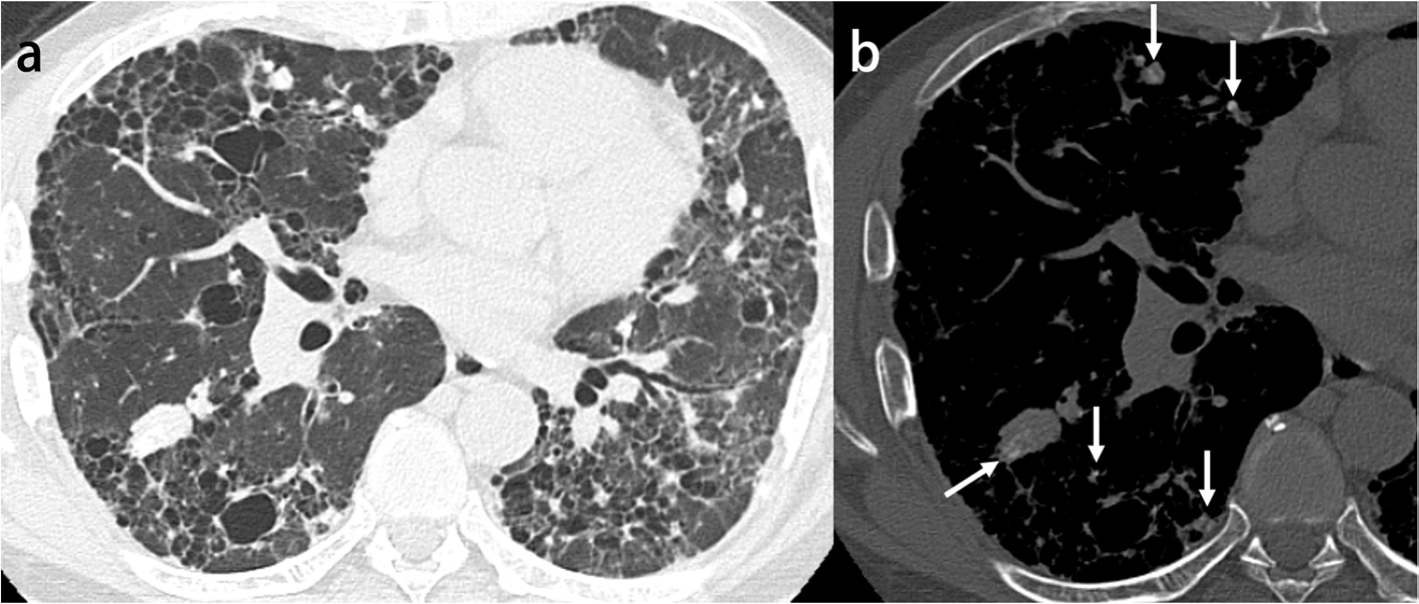
Pulmonary hyalinizing granuloma in a 47-year-old man. **a** CT image shows multiple bilateral irregular well-circumscribed nodules. Cyst formation is also seen. **b** Bone window image shows calcification in the nodules (arrows)

The nodular lesions of PHG are classically described as well-defined solitary or multiple pulmonary nodules or masses. They may have irregular margins without preference for any particular parenchymal location. In their review, Lhote et al. reported that calcification was found in 6 of 138 cases (4%). Most of the PHG nodules or masses were between 1.5 and 5 cm in size (Fig. 6) [29]. There have been reports of cyst and cavity formation, which could be caused by ischemic necrosis [30].

##### Allergic bronchopulmonary aspergillosis

Allergic bronchopulmonary aspergillosis (ABPA) is an immunological lung disorder caused by hypersensitivity to *Aspergillus fumigatus*. An allergic reaction to *Aspergillus* antigens is responsible for a local inflammatory reaction, such as infiltration of eosinophils, excessive mucus production, and bronchial wall damage.

**Fig. 7:**
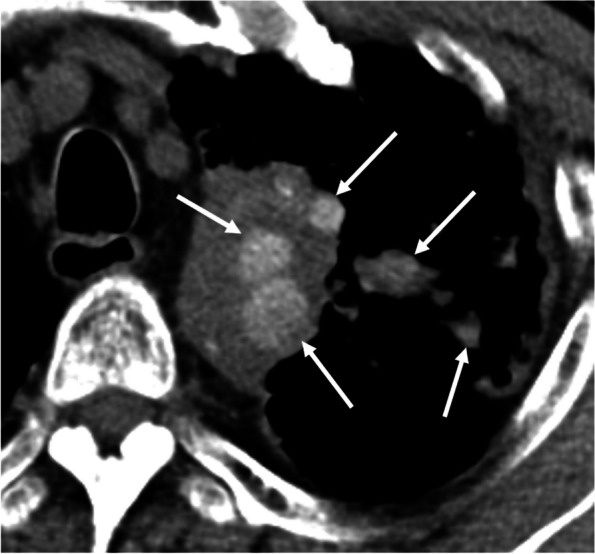
Allergic bronchopulmonary aspergillosis in a 62-year-old man. CT image shows high-attenuation mucus plugs in the left upper lobe (arrows)

Typically, CT reveals central bronchiectasis, usually involving segmental or subsegmental bronchi, with upper lobe predominance [31]. Bronchiectasis filled by mucoid impactions with an inversed Y or V shape is called finger-in-glove opacity [32]. High-attenuation mucus, characterized by higher attenuation than paraspinal muscles, is a pathognomonic finding of ABPA and can be seen in up to 20% of patients (Fig. 7) [33]. The cause of high-attenuation is explained by the presence of calcium salts and even metals, such as ions of iron and manganese, or desiccated mucus [31]. Furthermore, species of *Aspergillus* other than *A. fumigatus* and other filamentous fungi can cause similar pathologies called allergic bronchopulmonary mycosis [34], which is also characterized by a high-attenuation mucus plug [35].

##### Amyloidosis

Amyloidosis is a group of diseases resulting from the extracellular accumulation of abnormal protein in tissues and organs throughout the body. Three major subtypes of pulmonary involvement have been described: tracheobronchial, nodular parenchymal, and diffuse alveolar septal [36]. We provide an overview of nodular parenchymal and diffuse alveolar septal amyloidosis in this category, and a case of tracheobronchial amyloidosis is shown in Category 6.

Nodular parenchymal amyloidosis may manifest as solitary or multiple pulmonary nodules and mimic various diseases, from granulomatous disease to malignancy. Solitary tumor-like deposits, termed amyloidoma in the absence of systemic disease, are present in 60% of patients. Multiple pulmonary nodules are common and may exhibit smooth, lobulated, or spiculated margins [37], and calcification or ossification is seen in up to 50% of cases (Supplementary Fig. S6) [38, 39]. Alveolar septal pulmonary amyloidosis is characterized by well-defined 2–4-mm scattered micronodules accompanied by reticular opacities, interlobar septal thickening, and confluent consolidations with a basal and peripheral predominance [37].

In addition, the cysts may also be associated with calcified or noncalcified soft-tissue nodules. Nodular amyloidosis associated with thin-walled cysts is most common in patients with Sjögren syndrome. However, whether the cysts manifest lymphocytic interstitial pneumonia as a result of Sjögren syndrome or are a manifestation of amyloidosis, possibly due to small airway obstruction by amyloid deposits, is unclear [37, 40].

##### Silicosis

The inhalation of dust containing silica causes silicosis. It occurs in two distinct forms: acute silicosis, also known as silicoproteinosis, and classic or chronic silicosis, with the development of nodular infiltrative lung disease. Silicoproteinosis is a rare disease that can cause the rapid onset of respiratory failure following massive exposure to silica dust. Foci of calcification within areas of consolidation were seen in 83% of patients [41]. Classic or chronic silicosis typically manifests after 10 to 20 years of exposure to low concentrations of silica dust, with classic silicosis leading to lung fibrosis. The direct cytotoxic effects of silica cause macrophage death, followed by the release of inflammatory cytokines and other substances, and induce fibroblast proliferation [42].

**Fig. 8:**
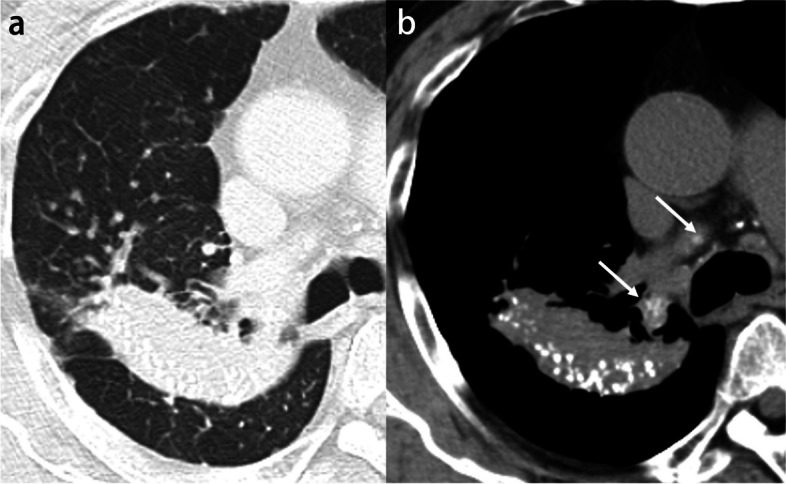
Silicosis (complicated form) in a 77-year-old man. **a** CT image shows a conglomerate mass, progressive massive fibrosis (PMF), in the right upper and lower lobes that crosses the major fissure. **b** Punctate and nodular calcifications are seen in the PMF. Mediastinal and hilar lymph nodes are also calcified (arrows)

Classic silicosis has two forms, simple and complicated, according to the radiographic findings. Simple silicosis is characterized on CT by the presence of multiple small nodules 2–5 mm in diameter with calcification. The nodules may be distributed diffusely throughout both lungs, but they tend to be most numerous in the upper lobes. On thin-section CT images, nodules are usually observed in centrilobular, paraseptal, and subpleural regions and show a perilymphatic distribution. Small nodular or branching centrilobular opacities may be an early sign of silicosis (Supplementary Fig. S7) [43]. Complicated silicosis, also termed progressive massive fibrosis, develops through the expansion and confluence of individual silicotic nodules. The most prominent HRCT finding of complicated silicosis is the development of large irregular nodules or masses or mass-like consolidation associated with apical scarring, distortion of lung architecture, and the development of adjacent bullae. These masses often develop in the middle portion or periphery of the upper lung zones and migrate toward the hila over time (Fig. 8). Calcification within conglomerate masses is also common [42]. Calcification of lymph nodes is common and typically occurs at the periphery of the node, termed eggshell calcification, which is characteristic, but punctate calcification has been reported as being more frequent [43].

### Step 4: Ascertain the predominant distribution in the upper or lower lungs

For Step 4, high-attenuation lesions without nodular or mass-like components can be further categorized into two groups, those in the upper and lower lungs, according to the presence of their predominant distribution.

#### Category 4: Upper lungs predominance

Metastatic calcification and pleuroparenchymal fibroelastosis (PPFE)-like lesion are included.

##### Metastatic calcification

**Fig. 9:**
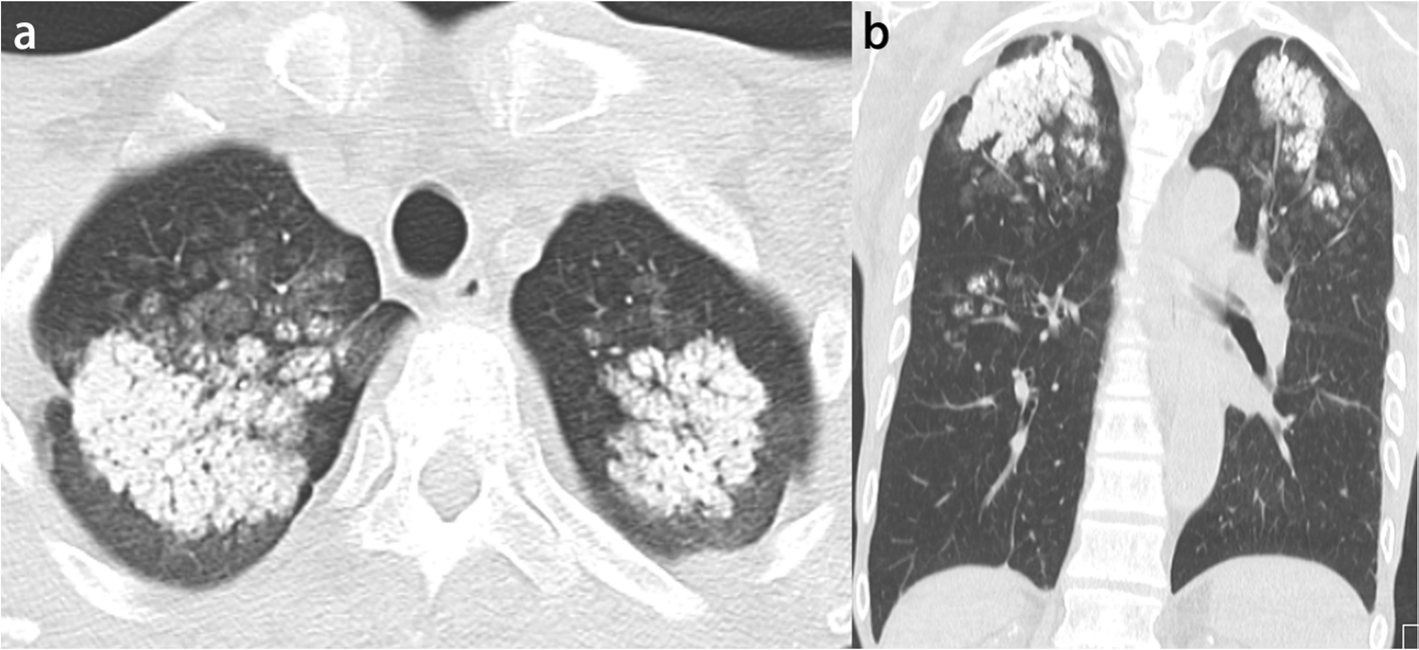
Metastatic calcification in a 61-year-old man. **a** CT image shows mass-like opacities containing dense calcification in the bilateral upper lobes. **b** Coronal CT image reveals that calcification is predominantly distributed in the upper lobes

Metastatic calcification is calcium deposition in normal lung tissue without prior tissue damage and is related to chronically elevated serum calcium phosphate product. The most common cause of metastatic calcification is hemodialysis for chronic renal insufficiency. Its prevalence in an unselected population was relatively low: only 14 cases (0.2%) of metastatic calcification were identified in a retrospective review of over 7000 autopsies [44]. In contrast, metastatic pulmonary calcification was found in 60–75% of hemodialyzed patients at autopsy [45, 46]. Calcium salts precipitate in an alkaline environment. The secretion of free hydrogen ions is an important local factor in developing metastatic calcification. The lungs, stomach, and kidneys secrete free hydrogens ions, resulting in an alkaline tissue environment. Furthermore, the pH of the blood in the lung is more alkalotic than that in other organs due to CO_2_ removal. Thus, the upper lobe predilection of pulmonary calcific disorders is explained by a higher blood pH and lower PaCO2 at the apex compared with the relatively lower pH at the base (Fig. 9). This difference is accounted for by the higher ventilation-perfusion ratio at the lung apex compared with the base [47].

**Fig. 10:**
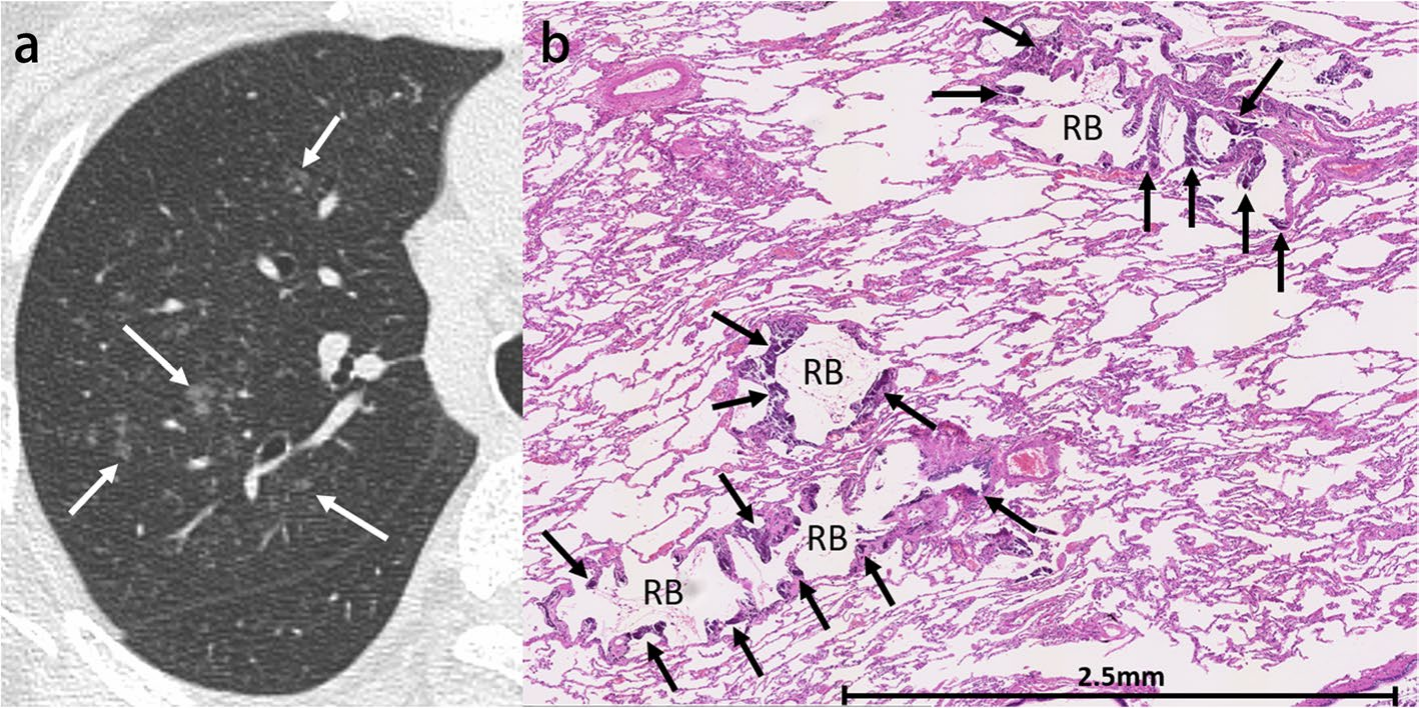
Metastatic calcification in a 70-year-old woman. **a** CT image shows poorly defined nodular opacities in the right upper lobe (arrows). **b** Photomicrograph (H-E stain) reveals calcification around respiratory bronchioles (arrows). RB, respiratory bronchiole

Radiologically, three patterns are identified: diffuse or patchy areas of ground-glass opacity or consolidation, multiple diffuse calcified nodules, and confluent high-attenuation consolidation with a predominantly lobar distribution (Supplementary Figs. S8 and S9) [48, 49]. Further, numerous fluffy and poorly defined nodules measuring 3–10 mm in diameter, with centrilobular ground-glass opacities, are also typical findings [50]. However, if the amount of calcium salts deposition is not high, such nodules may not be recognized as a high-attenuation lesion in mediastinal windows. Therefore, ground-glass opacity seen in lung windows may also reflect calcification. Only a micronodular or nodular high-attenuation lesion, corresponding to part of the lesion of metastatic calcification, may be identified in the mediastinal windows. In addition, calcification is often present in the vessels of the chest wall on CT scans (Supplementary Fig. S9) [48]. Pathologically metastatic calcification is characterized by strong hematoxylin staining in the alveolar space, alveolar septa, and the walls of small pulmonary vessels and bronchi (Fig. 10) [51].

##### Pleuroparenchymal fibroelastosis-like lesion

PPFE-like lesion presents as dense subpleural consolidation associated with the evidence of fibrosis in the upper lobe without differentiation between PPFE and the apical cap. According to Sumikawa et al., PPFE-like lesion was observed in 397 of 1284 patients (30.9%). In that study, old tuberculosis (TB) and chronic airway diseases strongly correlated with the presence of PPFE-like lesion, indicating that repeated inflammatory damage might cause such a lesion [52]. Small calcified nodules with soft tissue attenuation can be identified on CT (Supplementary Fig. S10).

#### Category 5: Lower lungs predominance

This category includes pulmonary alveolar microlithiasis, pulmonary ossification, lipiodol embolism, talc pleurodesis, and thoracolithiasis.

##### Pulmonary alveolar microlithiasis

Pulmonary alveolar microlithiasis is a rare autosomal recessive disease. The mutation of the *SLC34A2* gene prevents the movement of phosphorus ions from the alveolar spaces into type II pneumocytes, thus leading to the formation of calcium phosphate microliths in the alveolar spaces [53]. The microliths are round or ovoid with a concentric lamellated appearance and range from 0.01 to 2.8 mm in diameter [54].

**Fig. 11:**
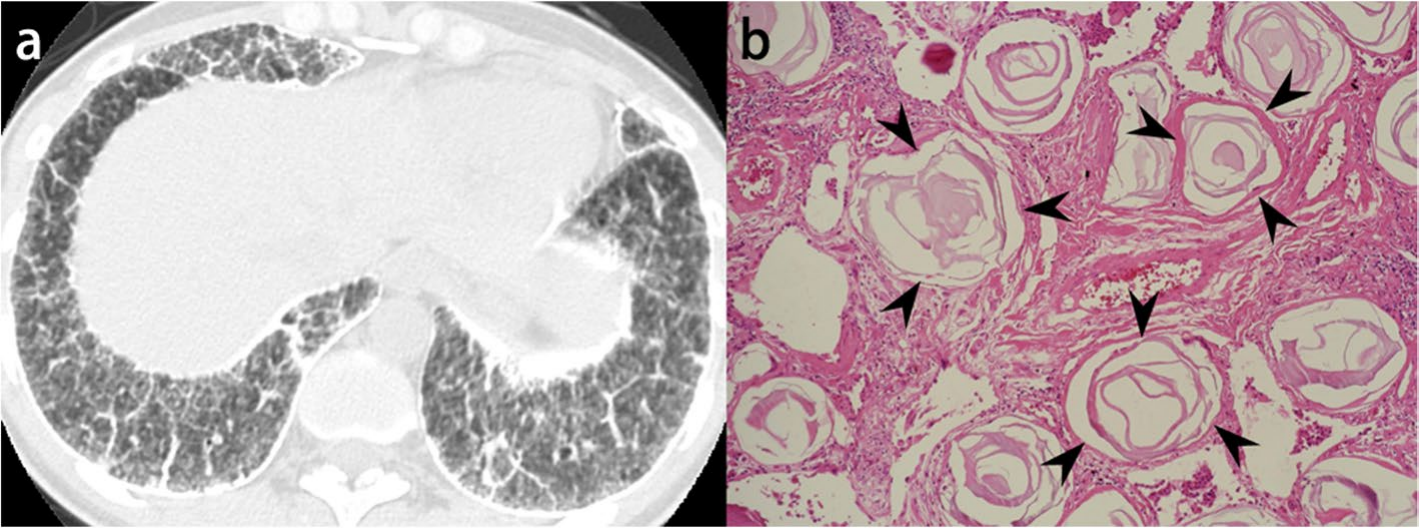
Pulmonary alveolar microlithiasis in a 39-year-old woman. **a** CT image shows extensive calcification with interlobular septa predominance. **b** Photomicrograph (original magnification, × 8; decalcified H-E stain) reveals concentrically lamellated calcified bodies in the intra-alveolar spaces (arrowheads)

The most common findings on HRCT are diffuse ground-glass opacities and small calcified parenchymal nodules, which preferentially involve the posterior, inferior, and central regions of the lungs. Other CT features include calcifications along the interlobular septa, bronchovascular bundles, fissures, and pleura (Fig. 11). Dense areas of consolidation and subpleural cysts that represent dilated alveolar ducts [55] may also be identified. The small calculi within the alveolar spaces result in diffuse ground-glass opacities, and the presence of high-attenuation micronodules is called “sandstorm appearance”. In addition, calcifications along the interlobular septa lead to linear opacity, and these calculi can converge to form larger parenchymal nodules or more confluent consolidations.

##### Pulmonary ossification

**Fig. 12:**
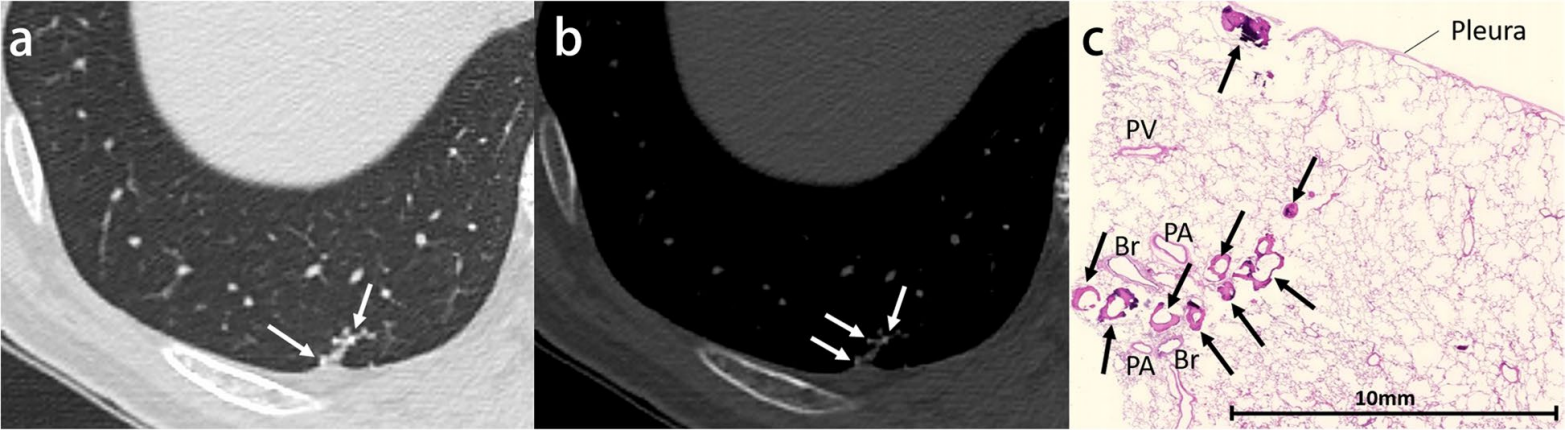
Idiopathic dendriform pulmonary ossification in a 76-year-old man. **a** CT image shows tiny contiguous branching nodules in the subpleural lesion of the right lower lobe (arrows). **b** Bone window image shows high attenuating small nodules (arrows). **c** Photomicrograph (H-E stain) reveals multiple bone formations in the alveolar ducts and alveoli (arrows). Br, bronchiole; PA, pulmonary artery; PV, pulmonary vein

**Fig. 13:**
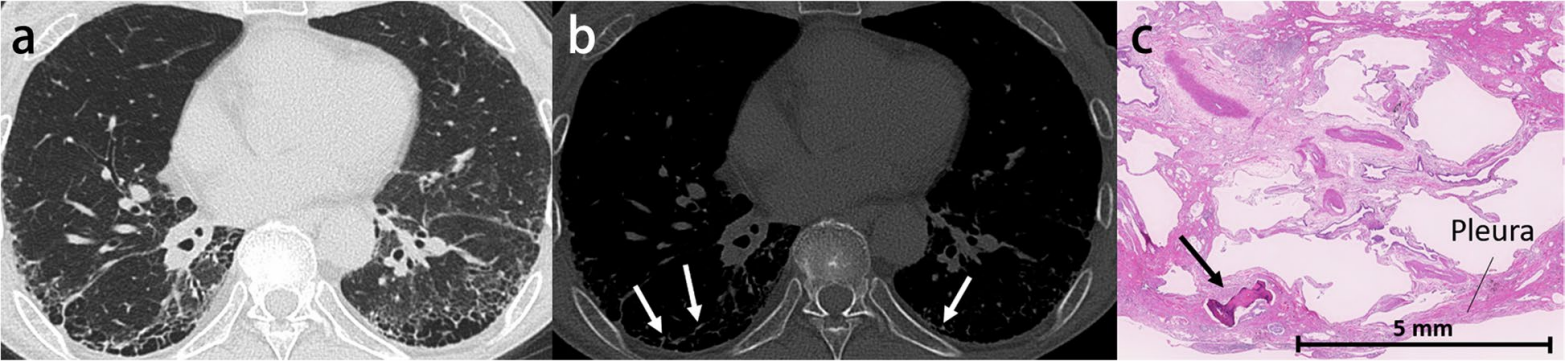
Secondary diffuse pulmonary ossification associated with systemic sclerosis-related interstitial lung disease in a 65-year-old woman. **a** CT image shows reticulation with cystic formation in the peripheral and bilateral lower lobes. **b** Bone window image shows high-attenuation nodules in the area of reticulation (arrows). **c** Photomicrograph (H-E stain) shows the tiny focus of ossification in the subpleural fibrotic lesion (arrow)

Pulmonary ossification is a rare condition that consists of bone formation in the alveolar spaces. Although the exact definition has not yet been established, widespread or extensive pulmonary ossification is called diffuse pulmonary ossification (DPO). According to autopsy examinations, the incidence of DPO is 1.63 in 1000 cases [56]. Pulmonary ossification can be idiopathic (Fig. 12) or secondary, resulting from conditions such as pulmonary congestion (particularly with mitral stenosis), aspiration, severe lung injury (Supplementary Fig. S11), and fibrosing interstitial lung disease (Fig. 13) [57–59]. Morphologically, pulmonary ossification is classified into two subtypes: dendriform and nodular type. Idiopathic cases are basically dendriform and show branching structures on CT [60]. Secondary cases can be both, and those associated with interstitial lung disease often appear as tiny nodules on imaging, even if they are dendriform on histopathology, making morphological separation difficult. Bony spines are considered to cause a rupture of the elastic fiber layer of the visceral pleura, which may lead to pneumothorax [61].

The distribution of pulmonary ossification is predominantly in the lower lobe, with a predilection for the posterior and lateral basilar segments and lung bases. The posterior aspect of the upper lobes may also be involved; however, ossification in this area always occurs in the presence of concomitant lower lobe diseases [62]. Egashira et al. also reported that DPO associated with fibrosing interstitial lung disease was present in the peripheral lung, especially in areas of reticulation [57]. High attenuation, reflecting underlying ossification, can be identified in some but not all nodules. Thin-section and volume-rendering CT images are helpful in the detection of small high-attenuation foci (Supplementary Fig. S12) [62]. The use of bone windows rather than mediastinal windows makes multiple tiny ossifications easier to identify.

##### Lipiodol embolism

**Fig. 14:**
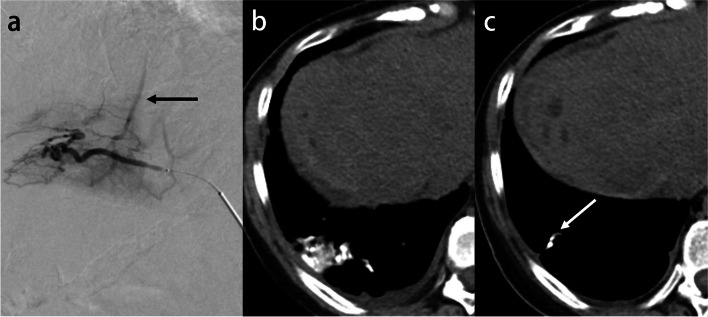
Pulmonary lipiodol embolism following transcatheter arterial chemoembolization for hepatocellular carcinoma in a 54-year-old woman. **a** A shunt between the right inferior phrenic artery and the hepatic vein (arrow) was revealed during chemoembolization with lipiodol. **b** Postoperative CT image shows a high-attenuation lesion in the right lower lobe, which is compatible with the accumulation of lipiodol. **c** Three months later, the dense lesion was smaller and had changed into a linear-like opacity (arrow)

Lipiodol is an ethyl ester of iodized fatty acids of poppy seed oil. Approximately 2% of patients who underwent percutaneous transcatheter arterial chemoembolization (TACE) developed pulmonary oily embolism. The lesions were found in all cases in the lower lobes [63], which may be due to the proximity to the treatment site of the liver. The risk factors for pulmonary lipiodol embolism after TACE include the dose of the iodized oil, the presence of an arteriovenous shunt, and trans-inferior phrenic artery embolization [64, 65]. ARDS may sometimes develop due to chemical injury caused by free fatty acid components [66]. Pulmonary oily embolisms were revealed within 12 to 48 h after TACE, and imaging abnormalities returned to normal between 12 and 35 days after the procedure [63]. In the early stages of the disease, in addition to deposits of high-attenuation lipiodol, the lung window shows ground-glass opacity reflecting hemorrhage resulting from lipiodol-induced infarction. Subsequently, phagocytosis by macrophages and lymphatic drainage may cause the lesions to shrink or resolve (Fig. 14).

##### Talc pleurodesis

**Fig. 15:**
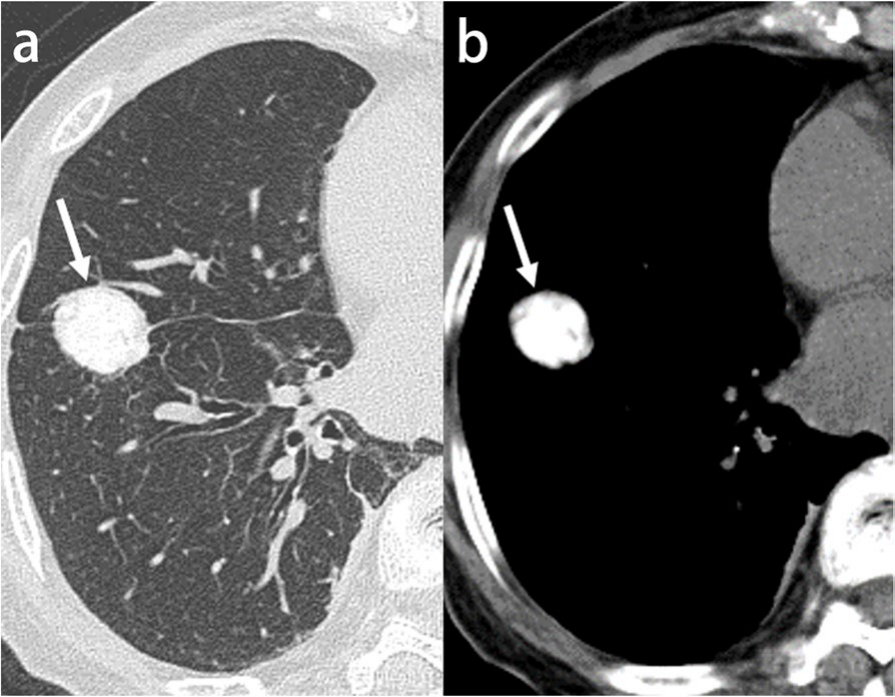
Talc pleurodesis in an 81-year-old woman. **a**, **b** CT images show a nodular high-attenuation talc deposit in the interlobar fissure (arrow)

Talc is a sclerosing agent for pleural adhesions and has been used in managing patients with benign and malignant pleural effusions and spontaneous pneumothorax. The presence of high-attenuation areas in the posterior basal regions of the pleural space is a typical finding after talc administration. However, it should be differentiated from findings of previous asbestos exposure, healed empyema, and TB. Talc deposits in the interlobar fissures with associated thickening of the fissure, which can possibly be mistaken for a calcified lung nodule, may also be encountered (Fig. 15) [67].

##### Thoracolithiasis

Thoracolithiasis is an entity in which one or more calcified or non-calcified loose bodies move freely in the thoracic cavity without any preceding trauma, intervention, or pleurisy. The prevalence of thoracolithiasis is approximately 0.086%. Thoracolithiasis usually manifests as a single calcified nodule and tends to occur in the left hemithorax. Most thoracoliths are located in the lower part of the pleural cavity, notably below the level of the top of each hemidiaphragm dome, which may be due to the effect of gravity. The longest diameter of thoracoliths ranges from 2.1 to 10.6 mm, and they all show calcification with an oval or round-shape. Because of the continuous movement associated with breathing, thoracoliths can become rounded and polished. Based on the presence of a necrotic fatty core in most thoracoliths and their left-sided predominance, dropping of twisted or torn epipericardial fat undergoing aseptic necrosis seems to be the most explainable etiology. As the mobility of the nodule is the critical finding in the diagnosis, comparison with older imaging studies is crucial (Supplementary Fig. S13) [68].

### Step 5: Identify the morphological pattern: linear, consolidation, nodular, or micronodular

If there is no distributional predominance, Step 5 is the final step. High-attenuation abnormalities are classified into linear, consolidation, nodular, or micronodular pattern according to morphological characteristics.

#### Category 6: Linear pattern

Linear high-attenuation lesions include staple line, tracheobronchial calcification, and granulomatous infection. Among these lesions, staple line is often difficult to distinguish from granulomatous infection, and tracheobronchial calcification may be misdiagnosed as calcified lesions of non-bronchial origin.

##### Tracheobronchial calcification

**Fig. 16:**
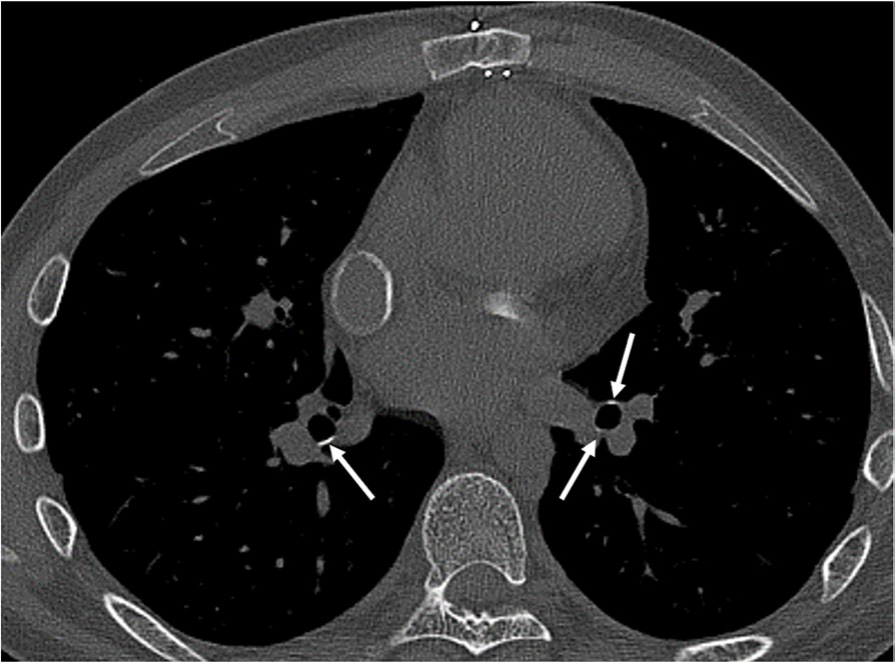
Tracheobronchial calcification associated with long-term warfarin therapy after pulmonary artery banding and ligation of patent ductus arteriosus in a 27-year-old man. Bone window CT image shows that calcifications can be identified along the bronchi (arrows)

Calcification of tracheobronchial cartilage has been recognized as an age-related phenomenon that occurs almost exclusively in patients over 40 years old. Compared to age-matched control subjects, an increased incidence of tracheobronchial calcification in adults receiving warfarin sodium was present. Several case reports of tracheobronchial calcification in pediatric patients with a history of postoperative cardiac surgery have also been published (Fig. 16) [69–71]. Warfarin is thought to be responsible for forming tracheobronchial calcification based on the hypothesis that warfarin inhibits the normal formation of a vitamin K-dependent protein that prevents calcification of cartilage and connective tissue [72].

Tracheobronchial calcification may also occur in tracheobronchopathia osteochondroplastica, relapsing polychondritis, and tracheobronchial amyloidosis (Supplementary Fig. S14) accompanied by wall thickening. Tracheobronchopathia osteochondroplastica and relapsing polychondritis spare the posterior membranous wall and affect only the cartilaginous portion. In tracheobronchial amyloidosis, however, involvement is circumferential, thus affecting the posterior membranous wall as well [73].

##### Staple line

In recent years, surgical staples, commonly made from titanium [74], have come into widespread use in thoracic surgery, especially in video-assisted thoracic surgery. Surgical staples are depicted as high-attenuation linear opacities. Nevertheless, in the case of partial resection of a lung lobe, the resection margin of the bronchus may not be identifiable, making it difficult to determine that it is a postoperative change. In addition, in cases of postoperative changes such as minor collapse or post-inflammatory scarring around the staple line, it is difficult to distinguish staple lines from calcified lesions, including old pulmonary TB, which presents as linear high-attenuation lesions. Furthermore, although surgical staples are metallic, their sizes are small and they often do not present as metallic artifacts, making them difficult to identify as staple lines. However, staple lines are depicted as a continuous, linear, high-attenuation structure always attached to the pleura. Besides, a linear structure can be differentiated from other diseases by the presence of a continuous high-attenuation nodular opacity, which corresponds to the surgical staples (Supplementary Fig. S15).

#### Category 7: Consolidation pattern

Amiodarone pulmonary toxicity and metastatic calcification may show high-attenuation consolidation. However, metastatic calcification is found predominantly in the upper lungs and is therefore described in Category 4.

##### Amiodarone pulmonary toxicity

Amiodarone, an anti-arrhythmic agent for refractory tachyarrhythmias, contains 37.3% iodine by weight. This high iodine content makes possible the detection of amiodarone deposits in the lung by CT [75]. The risk is highest in the first 6 to 12 months of treatment, and the frequency of onset increases as the cumulative dose increases from 101 to 150 g. Amiodarone and its metabolites accumulate in macrophages and type II pneumocytes in the lungs. Due to the very long half-life of amiodarone, the CT attenuation value increases. In roughly 90% of cases, elevated liver and spleen concentrations are also found, which can be identified through the lung bases on chest CT.

**Fig. 17:**
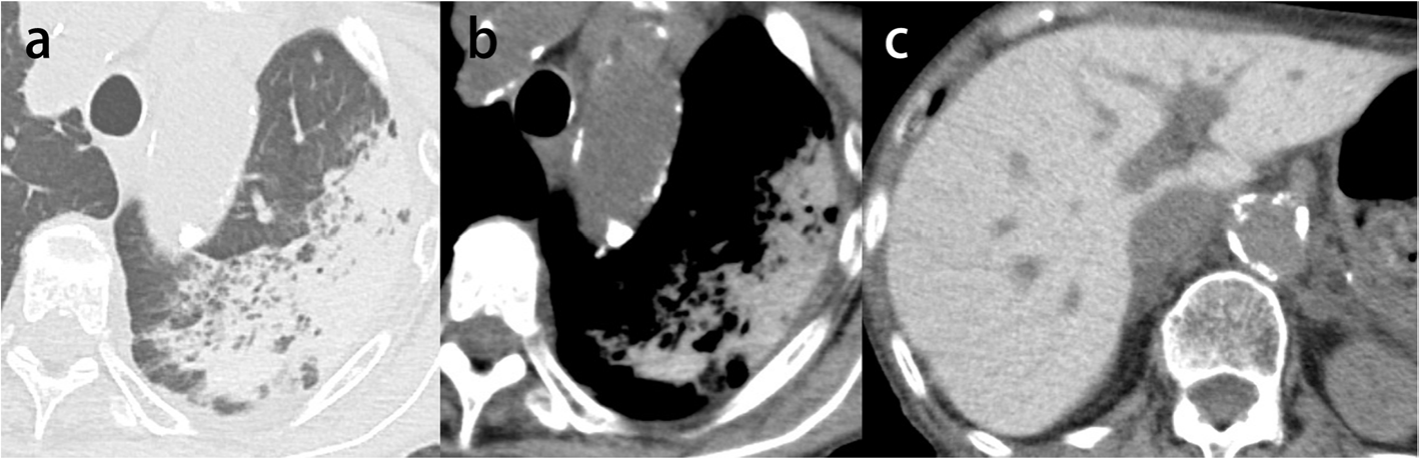
Amiodarone pulmonary toxicity in an 85-year-old woman. **a** CT image shows an extensive area of consolidation in the left upper lobe. **b** Mediastinal window image shows consolidation with high attenuation. **c** The liver shows increased attenuation, which is compatible with amiodarone deposition

The HRCT findings of amiodarone lung include ground-glass opacities, focal or diffuse areas of consolidation, and reticular opacities. However, the unique feature of amiodarone-related lung toxicity is the presence of foci of attenuation greater than that of the soft tissue due to tissue accumulation of this iodinated compound (Fig. 17) [76].

#### Category 8: Nodular pattern

As the Nomenclature Committee of the Fleischner Society has recommended that the term micronodule should be used to refer to nodules of less than 3 mm in diameter [77], we use the term “nodular pattern” to apply to cases that often show high-attenuation nodules of 3 mm or larger. Nodular pattern occurs in three distinct diseases: granulomatous infection, broncholithiasis, and hematoma. However, there may be overlaps between micronodular patterns, especially with granulomatous infection. The absorption process of a hematoma due to pulmonary laceration may also fit this pattern, although the finding may change and eventually resolve.

##### Granulomatous infection

Microbial infections that elicit a granulomatous tissue response can also result in a variety of thoracic dystrophic calcifications [47]. TB is a common cause of intrathoracic calcifications, especially in Asia and Africa. Apical and posterior segments of the upper lobes and superior segments of the lower lobes are often involved because of the high ventilation-perfusion ratio and decreased lymphatic drainage. As dystrophic calcifications are the sequelae of infection, patients with a prior infection are more common than those with newly diagnosed active disease. Therefore, CT findings of distortion of bronchovascular structures, bronchiectasis, emphysema, and fibrotic bands are frequently seen with calcification. Calcification shows numerous patterns related to fibrosis and granulomatous tissue, such as linear, nodular, micronodular, or even with nodular or mass-like soft tissue-attenuation components (Supplementary Figs. S16 and S17). The Ghon focus, a primary lesion of *Mycobacterium tuberculosis*, usually heals through development of a fibrous capsule around the focus of infection, which may show tiny calcification (Supplementary Fig. S18). Calcification of tuberculoma, a round or oval granuloma with a wall lined by granulomatous inflammatory tissue or encapsulated by connective tissue, is found in 20 to 30% of patients [78] and may show coarse calcification. Soft tissue attenuation can be accompanied by calcification, which is related to caseous necrotic material. In addition, when caseous necrosis occurs, robust phosphatase activity can take place within the necrotic centers, and subsequently, calcification is invariably found in the phosphatase-positive areas [47].

Other granulomatous infections can also cause calcification. Histoplasmomas may show numerous small and diffuse calcified pulmonary nodules. Although uncommon, coccidiomycosis may result in single or multiple dystrophic calcified nodules [47]. Further, diffuse, dense calcifications of 2 to 3 mm may occur in otherwise normal lungs as a sequela of varicella pneumonia [79].

##### Broncholithiasis

Broncholithiasis is defined as calcified or ossified material (broncholith) within the tracheobronchial tree and can be referred to as endobronchial, peribronchial, or transbronchial. Any focal process that results in dystrophic calcification can potentially lead to broncholithiasis. The most well-known theory of broncholithiasis is that necrotizing granulomas within the lymph nodes calcify and erode the bronchial wall, protruding into the tracheobronchial tree. In addition, necrotizing granulomas of TB can leak into the airway to facilitate transmission of the bacilli as a survival mechanism [80]. Further, mucus is a known cause of the endobronchial origin of broncholiths, and bronchiectasis is frequently reported as an underlying disease (Supplementary Fig. S19) [81].

The incidence of broncholiths was highest in the bronchus intermedius, followed by the left upper lobe and right upper lobe (Supplementary Fig. S20) [80]. Due to the anatomy of the airway and the distribution of the lymph nodes, broncholiths tend to occur more on the right side, especially in the upper and middle lobes [82]. Long-standing airway obstruction may lead to bronchiectasis, and air trapping and lobar or segmental atelectasis or consolidation may also occur. Endobronchial actinomycosis associated with broncholiths has also been reported [83].

##### Hematoma

**Fig. 18:**
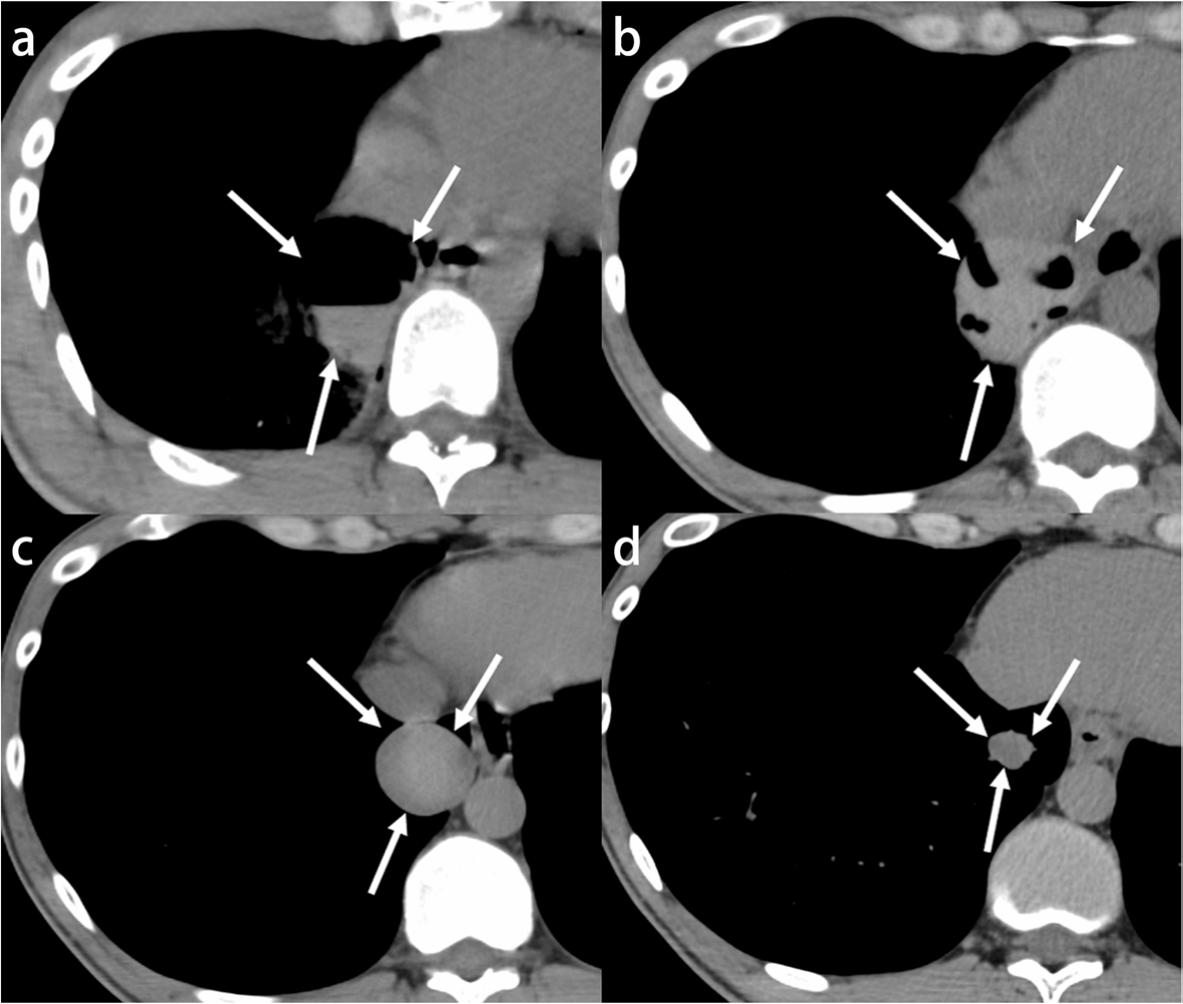
Pulmonary laceration due to traumatic injuries in a 28-year-old man. **a** CT image shows a high-attenuation air-fluid level in the right lower lobe (arrows). **b** The lacerated cyst has decreased in size, and the air bubble has been absorbed (arrows). **c** Although the air bubble has disappeared, the attenuation of the nodule remains high (arrows). **d** Attenuation of the nodule has decreased, and subsequent shrinkage of the nodule is shown (arrows)

Intrapulmonary hematomas are often present in the course of traumatic pulmonary lacerations. In the early stages of injury, a high-attenuation air-fluid level (hematopneumatocele) is characteristic. Subsequently, lacerations can take weeks to months to resolve as the air inside the laceration is gradually absorbed, eventually turning into a hematoma presenting as a solid, homogeneous, high-attenuation mass (Fig. 18). Hematomas have been found to shrink less than 0.5 cm in 3 weeks, but finally, the hematoma becomes a small fibrous nodule. The time to resolution of hematomas observed on imaging varies: while most resolve gradually within 5 weeks, some can take up to a year to resolve after injury [84].

Ehlers-Danlos syndrome subtype IV may cause spontaneous pulmonary laceration because of a defect of type III collagen. Following laceration, bleeding from disrupted alveolar walls and pulmonary vasculature results in the formation of hematoma [85].

#### Category 9: Micronodular pattern

Micronodular pattern is characterized by calcified nodules of less than 3 mm in diameter that result from granulomatous infection and cement migration.

##### Cement migration

Percutaneous vertebroplasty with acrylic cement (polymethyl methacrylate; PMMA) aims to prevent vertebral body collapse and pain in patients with pathologic vertebral bodies. As chest imaging is not routinely performed after this procedure, the true incidence of pulmonary cement embolism after percutaneous vertebroplasty is unknown. However, some studies reported an incidence of pulmonary cement embolism detected by chest radiograph and CT ranging from 4.6 to 6.8% [86, 87]. Venous leaks of PMMA significantly increase the risk of PMMA pulmonary emboli for the following reasons: the liquid consistency of the PMMA, the treatment of some malignant lesions in which more frequent cortical destruction of the vertebral body occurs, and high vascularity associated with some malignant tumors [86]. Solitary or multiple tubular or branching high-attenuation cement deposits within the pulmonary vessels are identified on CT (Supplementary Fig. S21).

## Limitations

The proposed diagnostic algorithm has limitations. First, there may be more than one category for the same disease. Second, diseases that are likely to be encountered in daily clinical practice were eligible for inclusion in this algorithm. Therefore, rare diseases, such as extramedullary plasmacytoma [88], synovial sarcoma [89], and hemosiderosis [12, 90] presenting with calcification, are not included. Despite these limitations, our proposed diagnostic algorithm is presented with a focus on diseases and patterns that are likely to be encountered in everyday clinical practice and provides a concise algorithmic method that can be practically useful for the differential diagnosis of high-attenuation pulmonary abnormalities.

## Conclusions

High-attenuation pulmonary abnormalities are caused by calcification, ossification, hemorrhage, iodine, and other etiologies. The majority of these abnormalities, such as granulomatous infections, are benign. However, they are also recognized in malignant diseases including lung cancers and metastatic tumors and can lead to a wide range of differential diagnoses. Some of these attenuations, such as hematomas and lipiodol embolisms, may change their morphology and characteristics over a relatively short time. Our proposed stepwise diagnostic algorithm for high-attenuation pulmonary abnormalities may help to recognize these imaging findings, to determine whether the associated diseases require further investigation, and to guide appropriate patient management.

### Supplementary Information

**Additional file 1: Supplementary Fig. S1.** Seed migration from prostate brachytherapy in an 88-year-old man. a CT image shows metallic artifact of a single seed that migrated to the right lower lobe (arrow). b Bone window image shows high punctate attenuation (arrow). **Supplementary Fig. S2.** Barium sulfate aspiration due to a history of nasopharyngeal carcinoma in a 55-year-old man. Bone window CT image shows barium sulfate with an obvious metallic artifact in the left lower lobe. Concurrent aspiration pneumonia is seen (arrows). **Supplementary Fig. S3.** Aspirated metallic dental implant in an 85-year-old man. a, b CT images show the metallic artifact of a dental implant that migrated to the right lower lobe bronchus (arrow). c Aspirated metal objects are easy to identify on the chest radiograph (arrow). **Supplementary Fig. S4.** Hamartoma in a 70-year-old man. a CT image shows a slightly lobulated nodule in the right lower lobe. b Mediastinal window image shows that the nodule contains focal calcification (arrows). Fat is absent. **Supplementary Fig. S5.** Metastatic papillary thyroid cancer in a 74-year-old man. CT image shows a soft tissue nodule with multiple nodular calcifications in the right upper lobe (arrow). **Supplementary Fig. S6.** Amyloidosis in a 74-year-old woman. Bone window CT image shows a mass along with the bronchovascular bundle with numerous irregular calcifications in the lingular segment (arrows), which is compatible with the nodular parenchymal type of amyloidosis. **Supplementary Fig. S7.** Silicosis (simple form) in a 76-year-old man. a CT image shows multiple small nodules with a perilymphatic (centrilobular and subpleural) distribution in both lungs. b Bone window image shows calcification in the nodules (arrows). **Supplementary Fig. S8.** Metastatic calcification in a 47-year-old man. CT image shows lobular ground-glass opacity with a centrilobular distribution. Focal dense calcification is also seen (arrows). **Supplementary Fig. S9.** Metastatic calcification in a 67-year-old man. Mediastinal window CT image shows dense calcification in the bilateral upper lobes. Note vascular calcification in the chest wall (arrows). **Supplementary Fig. S10.** Pleuroparenchymal fibroelastosis (PPFE)-like lesion in a 64-year-old male. Bone window CT image shows subpleural opacities with high punctate attenuation (arrows). Histopathology reveals calcification and ossification in the subpleural fibroelastosis. **Supplementary Fig. S11.** Secondary diffuse pulmonary ossification due to acute respiratory distress syndrome (ARDS) in a 75-year-old man. a CT image shows mixed coarse and fine reticulation with subpleural predominance in the left upper lobe. b Bone window image shows high-attenuation nodules in the area of coarse reticulation (arrows). High-attenuation nodules gradually increased during a 2-year period after suffering from ARDS. **Supplementary Fig. S12.** Idiopathic dendriform pulmonary ossification in a 65-year-old man. a CT image shows the lattice-like distribution of multiple linear and nodular opacities in the peripheral area of the lung bases. b Bone window CT image shows numerous high-attenuation small linear and branching structures (arrows). c Volume rendering image of the lungs. Bony lesions are shown as yellow color structures. d Magnified volume rendering image of the right lower lobe. Dendriform and coral-like shapes of bony structures are shown. **Supplementary Fig. S13.** Thoracolithiasis in a 50-year-old man. a, b Initial CT images show a smooth-marginated calcified nodule (arrow). c, d Follow-up CT images obtained eight years later show migration of the calcified nodule into the posteroinferior region (arrow). **Supplementary Fig. S14.** Tracheobronchial amyloidosis in a 22-year-old woman. CT image shows bronchial wall thickening with calcification (arrows). **Supplementary Fig. S15.** Postoperative status of pneumothorax in a 65-year-old man. Bone window CT image shows high-attenuation linear structures that abut the pleura, which correspond to surgical staples, are seen (arrows). **Supplementary Fig. S16.** Inactive tuberculosis in a 70-year-old man. CT image shows linear calcification in the right upper lobe, adjacent to the bulla (arrows). **Supplementary Fig. S17.** Inactive tuberculosis in a 90-year-old man. CT image shows numerous calcified nodules associated with soft tissues (arrows). **Supplementary Fig. S18.** Ghon focus in a 60-year-old man. CT image shows nodular calcification in the periphery of the right lower lobe (arrow). **Supplementary Fig. S19.** Broncholithiasis in a 58-year-old woman. Photomicrograph (original magnification, × 10; decalcified H-E stain) depicts a concentric decalcified stone that is considered to have originated from mucus. **Supplementary Fig. S20.** Broncholithiasis in a 71-year-old man. CT image shows a calcified nodule within a dilated bronchus near the left hilum (arrow). Calcified lymph nodes at the left hilum and mediastinum are also shown (arrowheads). **Supplementary Fig. S21.** Cement migration due to percutaneous vertebroplasty in a 77-year-old woman. a CT scout view shows post-multilevel vertebroplasty (arrows). b Postoperative bone window CT image shows cement migration to the left lower lobe (arrow).

## Data Availability

Not applicable.

## Abbreviations

ABPA: Allergic bronchopulmonary aspergillosis
ARDS: Acute respiratory distress syndrome
DPO: Diffuse pulmonary ossification
EHE: Epithelioid hemangioendothelioma
HRCT: High-resolution computed tomography
HU: Hounsfield unit
PHG: Pulmonary hyalinizing granuloma
PMMA: Polymethyl methacrylate
PPFE: Pleuroparenchymal fibroelastosis
PSP: Pulmonary sclerosing pneumocytoma
TACE: Transcatheter arterial chemoembolization
TB: Tuberculosis
