# Supplementary material for: Stepwise diagnostic algorithm for high-attenuation pulmonary abnormalities on CT
Source: Insights Imaging. 2023 Oct 20;14:177. doi: 10.1186/s13244-023-01501-x (PMC10587054; doi:10.1186/s13244-023-01501-x)
Supplement: Supplementary file 1 — Additional file 1: Supplementary Fig. S1. Seed migration from prostate brachytherapy in an 88-year-old man. a CT image shows metallic artifact of a single seed that migrated to the right lower lobe (arrow). b Bone window image shows high punctate attenuation (arrow). Supplementary Fig. S2. Barium sulfate aspiration due to a history of nasopharyngeal carcinoma in a 55-year-old man. Bone window CT image shows barium sulfate with an obvious metallic artifact in the left lower lobe. Concurrent aspiration pneumonia is seen (arrows). Supplementary Fig. S3. Aspirated metallic dental implant in an 85-year-old man. a, b CT images show the metallic artifact of a dental implant that migrated to the right lower lobe bronchus (arrow). c Aspirated metal objects are easy to identify on the chest radiograph (arrow). Supplementary Fig. S4. Hamartoma in a 70-year-old man. a CT image shows a slightly lobulated nodule in the right lower lobe. b Mediastinal window image shows that the nodule contains focal calcification (arrows). Fat is absent. Supplementary Fig. S5. Metastatic papillary thyroid cancer in a 74-year-old man. CT image shows a soft tissue nodule with multiple nodular calcifications in the right upper lobe (arrow). Supplementary Fig. S6. Amyloidosis in a 74-year-old woman. Bone window CT image shows a mass along with the bronchovascular bundle with numerous irregular calcifications in the lingular segment (arrows), which is compatible with the nodular parenchymal type of amyloidosis. Supplementary Fig. S7. Silicosis (simple form) in a 76-year-old man. a CT image shows multiple small nodules with a perilymphatic (centrilobular and subpleural) distribution in both lungs. b Bone window image shows calcification in the nodules (arrows). Supplementary Fig. S8. Metastatic calcification in a 47-year-old man. CT image shows lobular ground-glass opacity with a centrilobular distribution. Focal dense calcification is also seen (arrows). Supplementary Fig. S9. Metastatic calcification [file 13244_2023_1501_MOESM1_ESM.docx]

**Stepwise diagnostic algorithm for high-attenuation pulmonary abnormalities on CT**

**ELECTRONIC SUPPLEMENTARY MATERIAL**

**
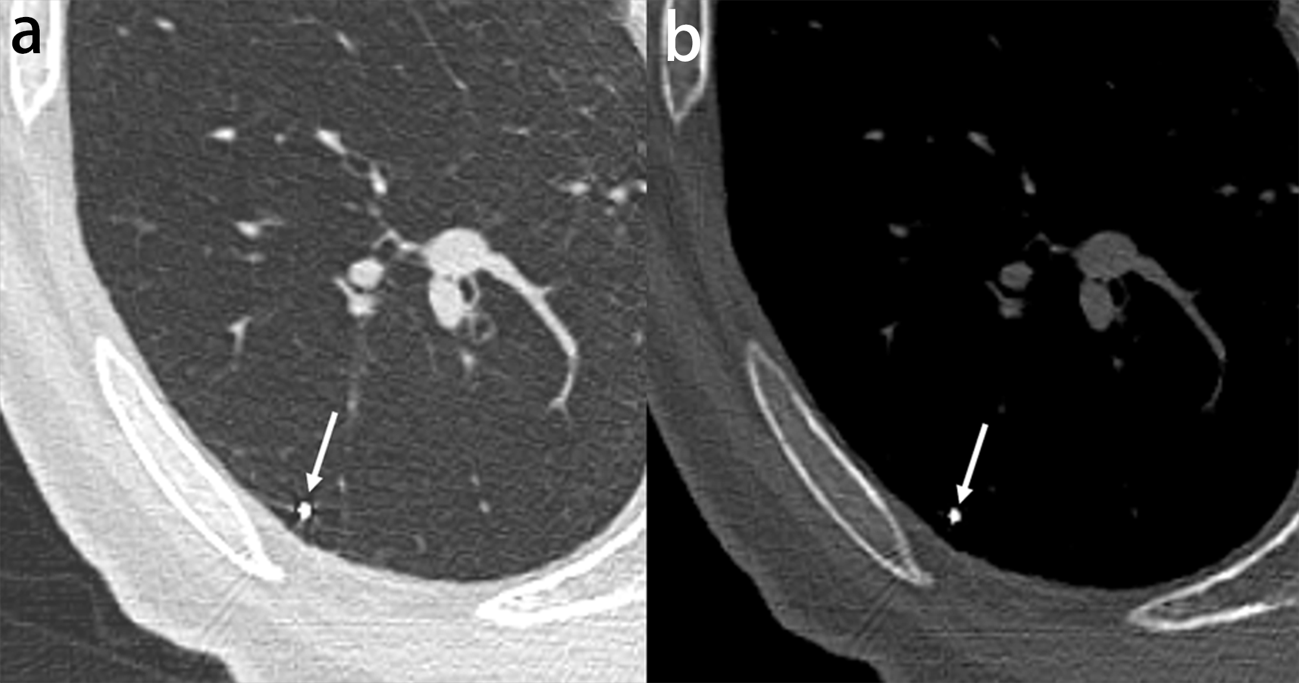
**

**Supplementary Fig. S1.** Seed migration from prostate brachytherapy in an 88-year-old man. **a** CT image shows metallic artifact of a single seed that migrated to the right lower lobe (arrow). **b** Bone window image shows high punctate attenuation (arrow)


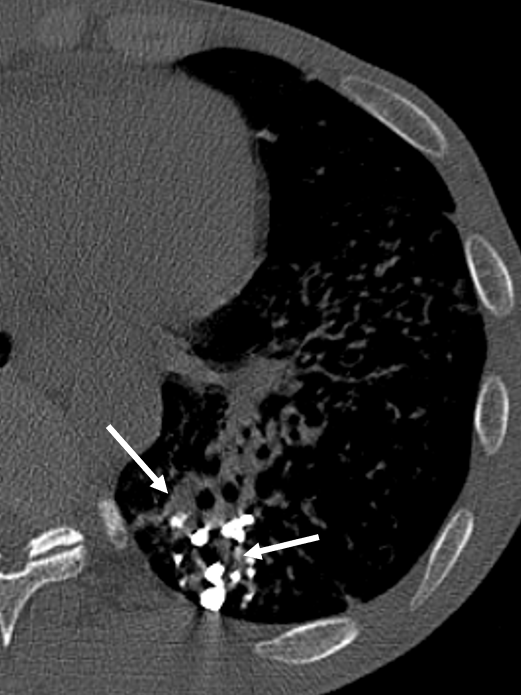


**Supplementary Fig. S2.** Barium sulfate aspiration due to a history of nasopharyngeal carcinoma in a 55-year-old man. Bone window CT image shows barium sulfate with an obvious metallic artifact in the left lower lobe. Concurrent aspiration pneumonia is seen (arrows)


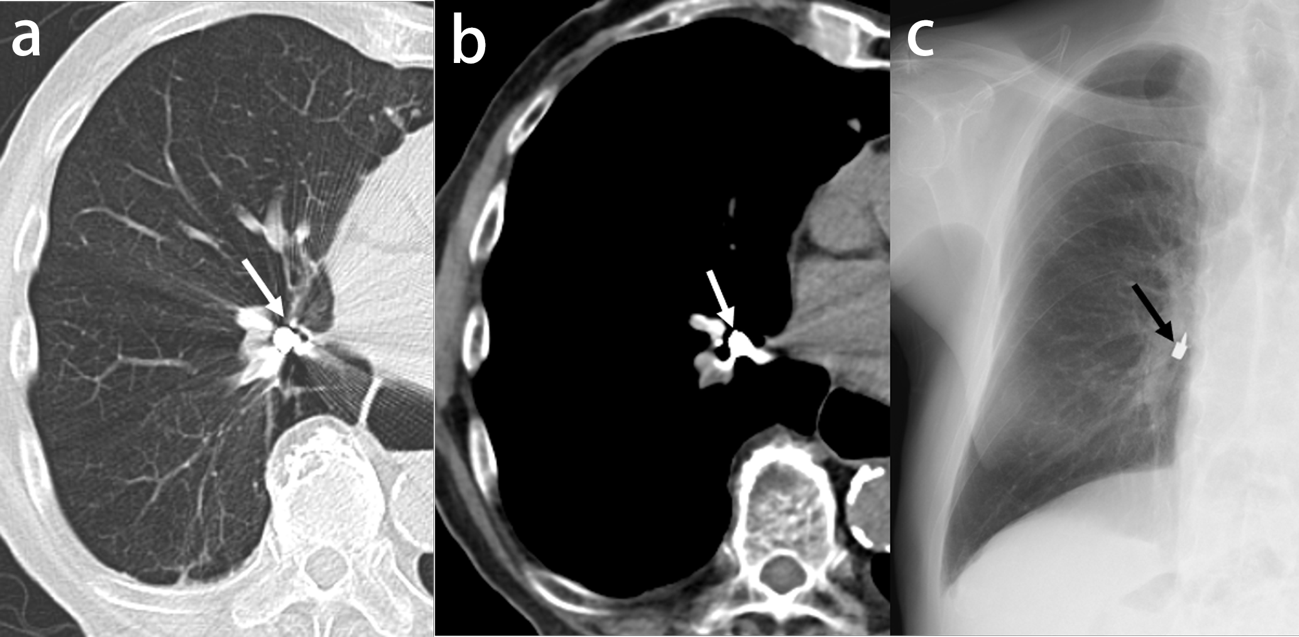


**Supplementary Fig. S3.** Aspirated metallic dental implant in an 85-year-old man. **a, b** CT images show the metallic artifact of a dental implant that migrated to the right lower lobe bronchus (arrow). **c** Aspirated metal objects are easy to identify on the chest radiograph (arrow)


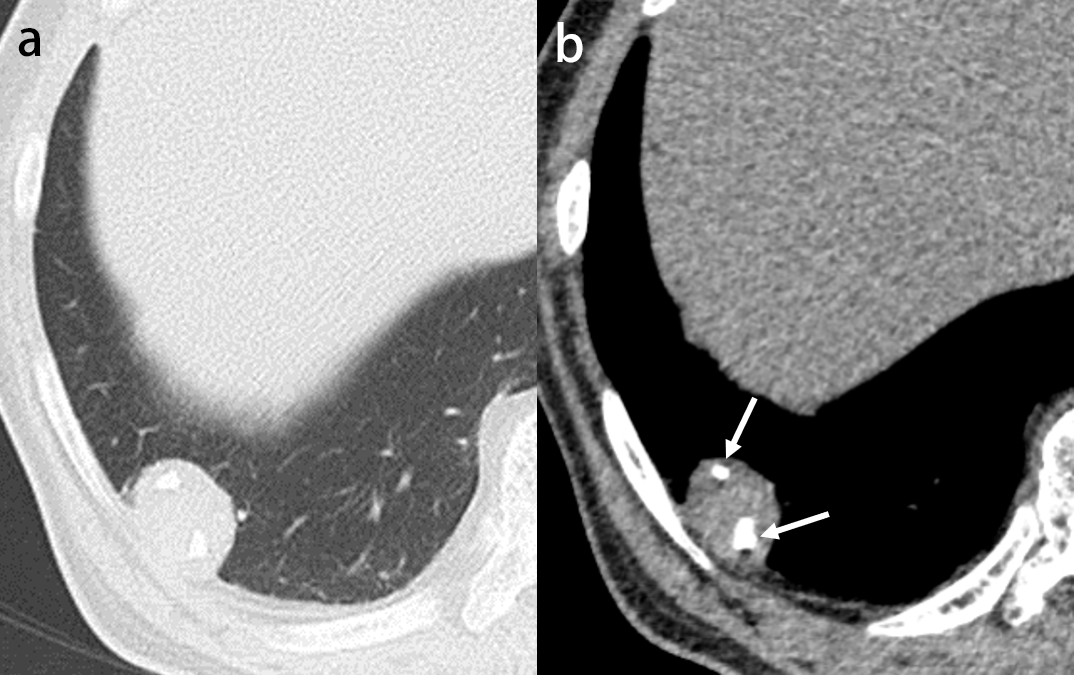


**Supplementary Fig. S4.** Hamartoma in a 70-year-old man. **a** CT image shows a slightly lobulated nodule in the right lower lobe. **b** Mediastinal window image shows that the nodule contains focal calcification (arrows). Fat is absent


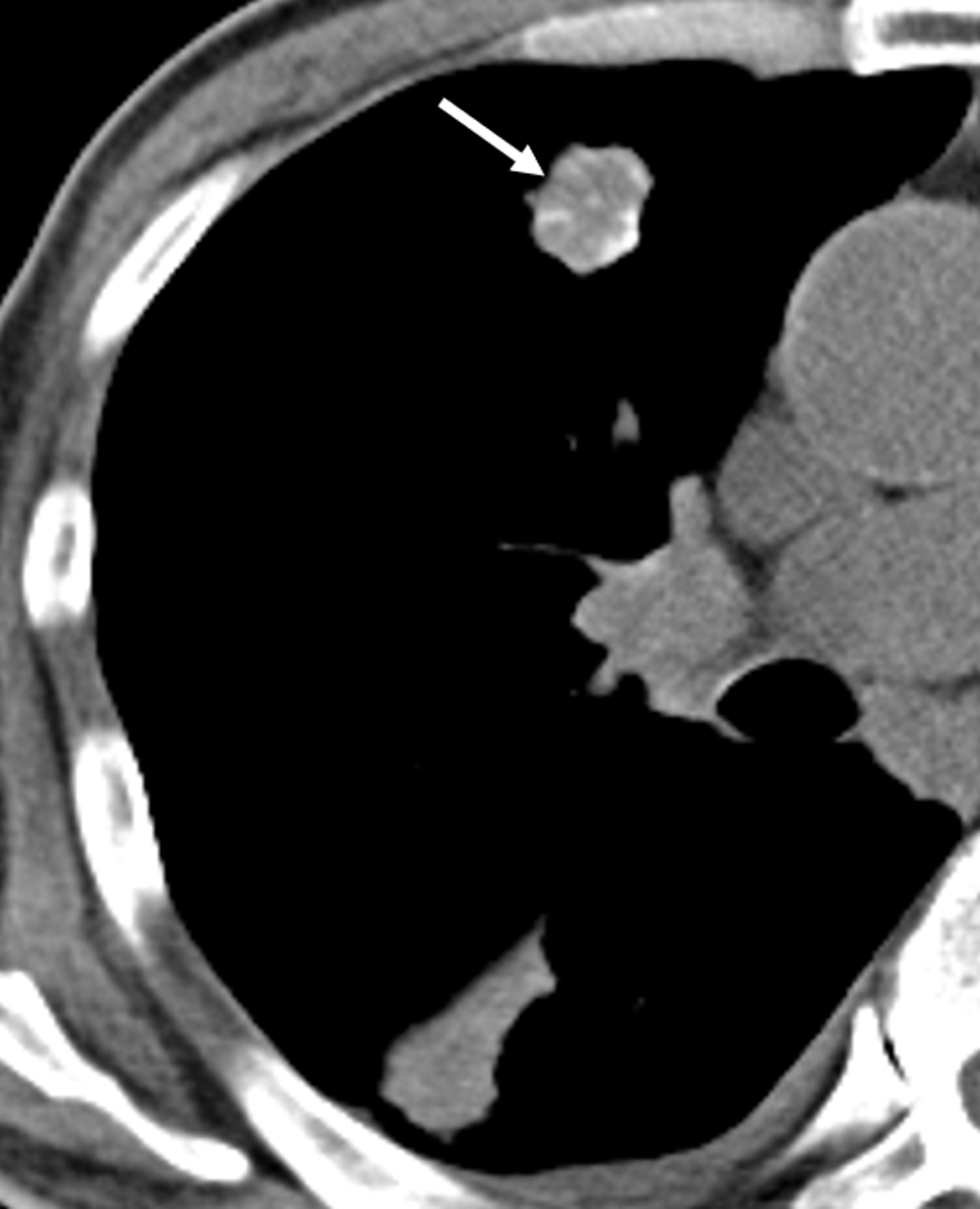


**Supplementary Fig. S5.** Metastatic papillary thyroid cancer in a 74-year-old man. CT image shows a soft tissue nodule with multiple nodular calcifications in the right upper lobe (arrow)


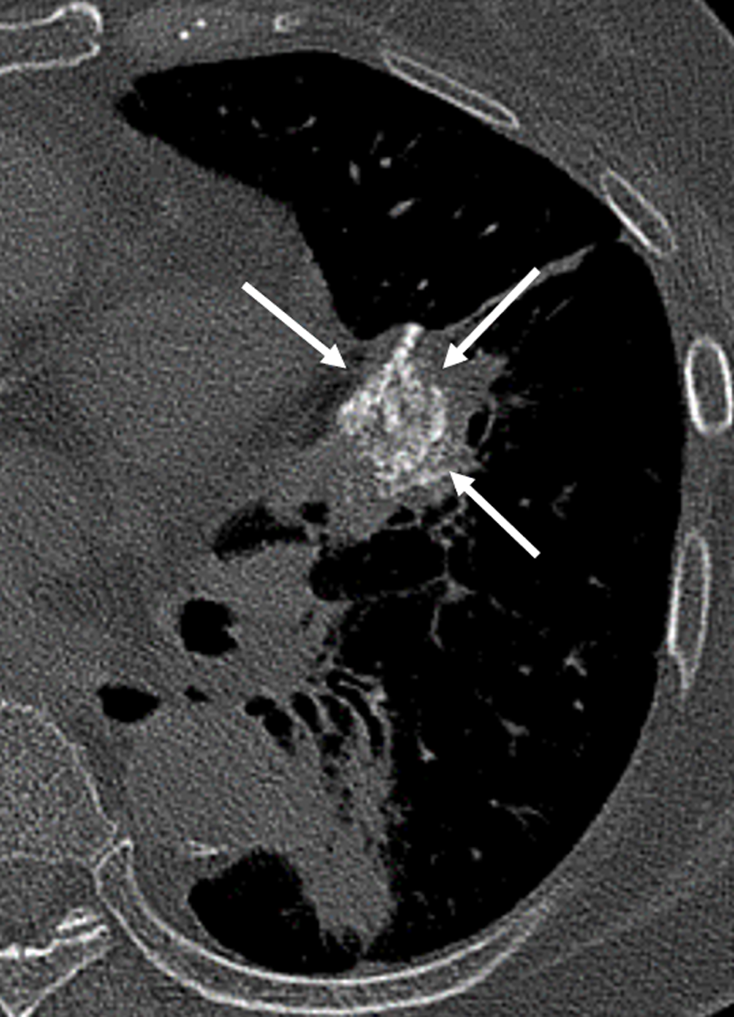


**Supplementary Fig. S6.** Amyloidosis in a 74-year-old woman. Bone window CT image shows a mass along with the bronchovascular bundle with numerous irregular calcifications in the lingular segment (arrows), which is compatible with the nodular parenchymal type of amyloidosis


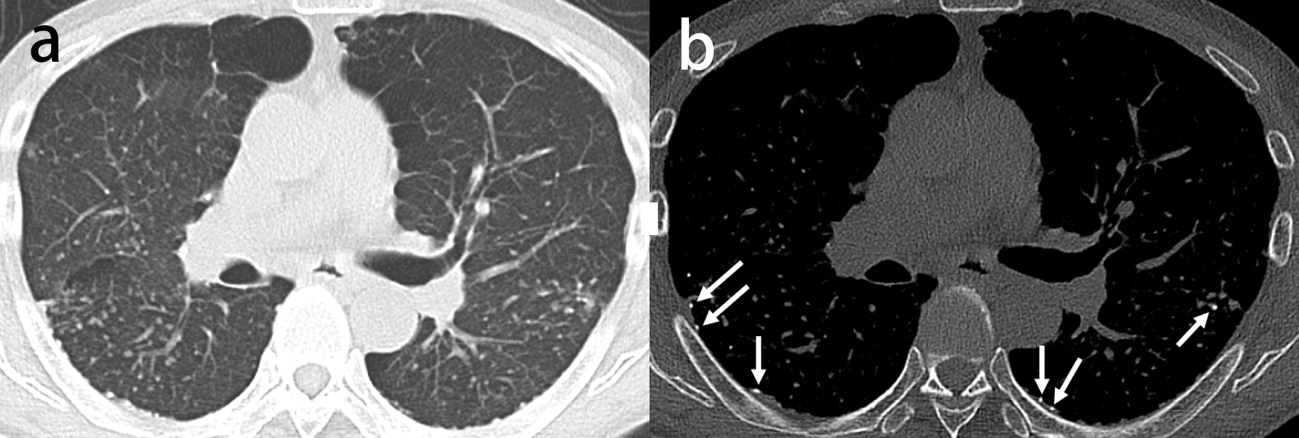


**Supplementary Fig. S7.** Silicosis (simple form) in a 76-year-old man. **a** CT image shows multiple small nodules with a perilymphatic (centrilobular and subpleural) distribution in both lungs. **b** Bone window image shows calcification in the nodules (arrows)


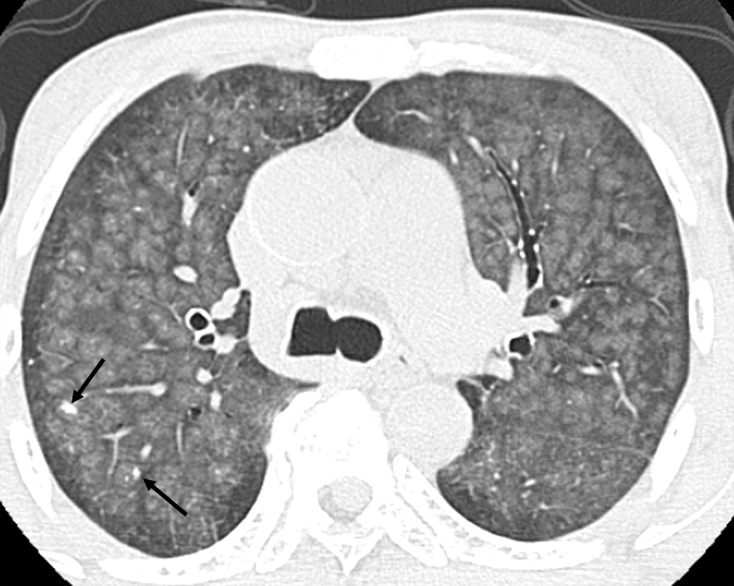


**Supplementary Fig. S8.** Metastatic calcification in a 47-year-old man. CT image shows lobular ground-glass opacity with a centrilobular distribution. Focal dense calcification is also seen (arrows)


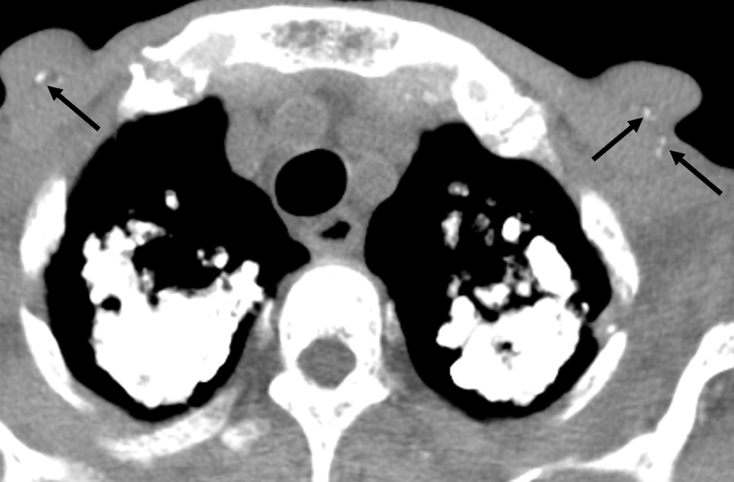


**Supplementary Fig. S9.** Metastatic calcification in a 67-year-old man. Mediastinal window CT image shows dense calcification in the bilateral upper lobes. Note vascular calcification in the chest wall (arrows)


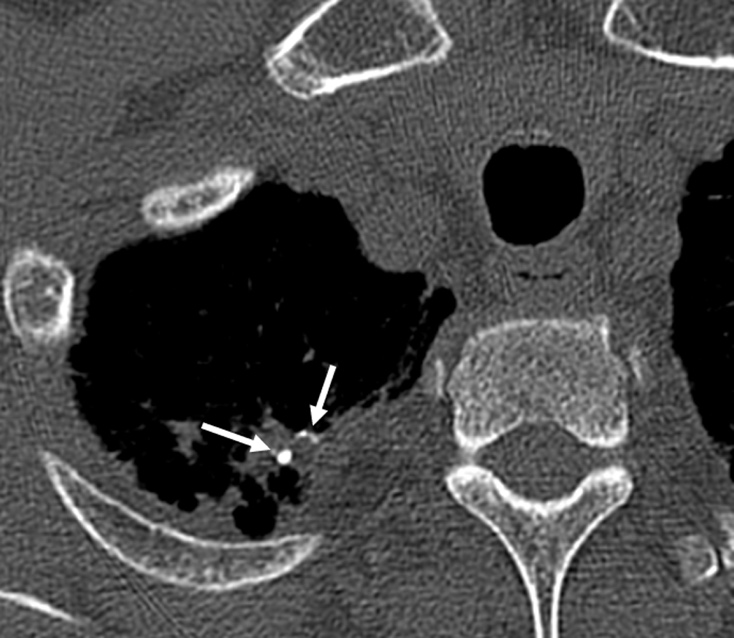


**Supplementary Fig. S10.** Pleuroparenchymal fibroelastosis (PPFE)-like lesion in a 64-year-old male. Bone window CT image shows subpleural opacities with high punctate attenuation (arrows). Histopathology reveals calcification and ossification in the subpleural fibroelastosis


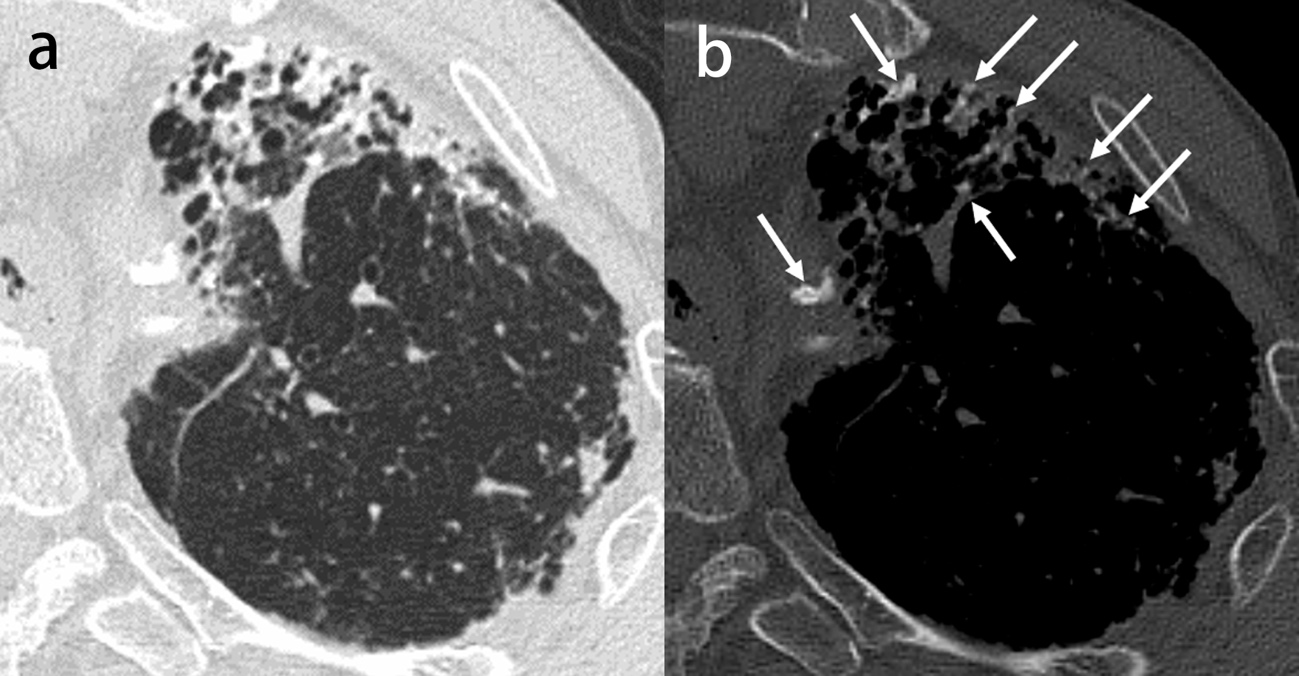


**Supplementary Fig. S11.** Secondary diffuse pulmonary ossification due to acute respiratory distress syndrome (ARDS) in a 75-year-old man. **a** CT image shows mixed coarse and fine reticulation with subpleural predominance in the left upper lobe. **b** Bone window image shows high-attenuation nodules in the area of coarse reticulation (arrows). High-attenuation nodules gradually increased during a 2-year period after suffering from ARDS


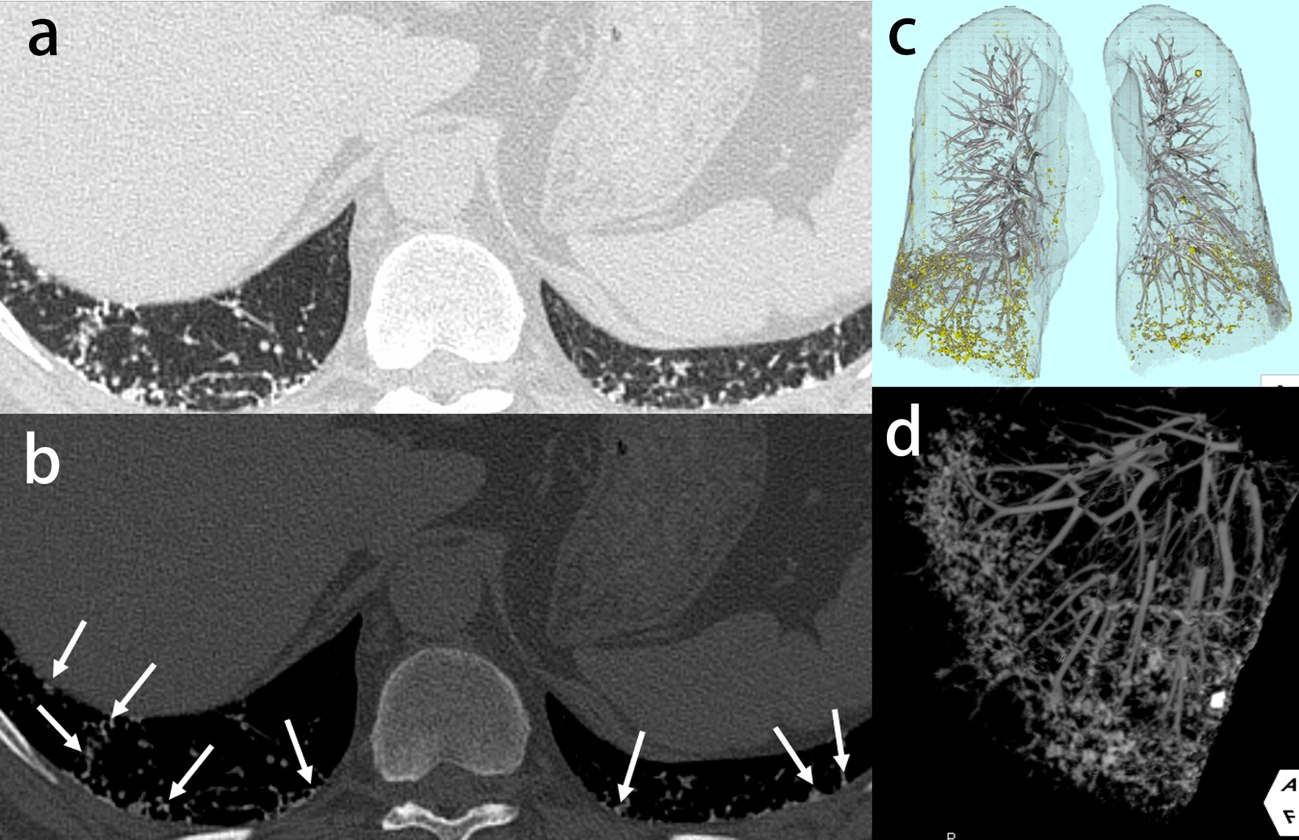


**Supplementary Fig. S12.** Idiopathic dendriform pulmonary ossification in a 65-year-old man. **a** CT image shows the lattice-like distribution of multiple linear and nodular opacities in the peripheral area of the lung bases. **b** Bone window CT image shows numerous high-attenuation small linear and branching structures (arrows). **c** Volume rendering image of the lungs. Bony lesions are shown as yellow color structures. **d** Magnified volume rendering image of the right lower lobe. Dendriform and coral-like shapes of bony structures are shown


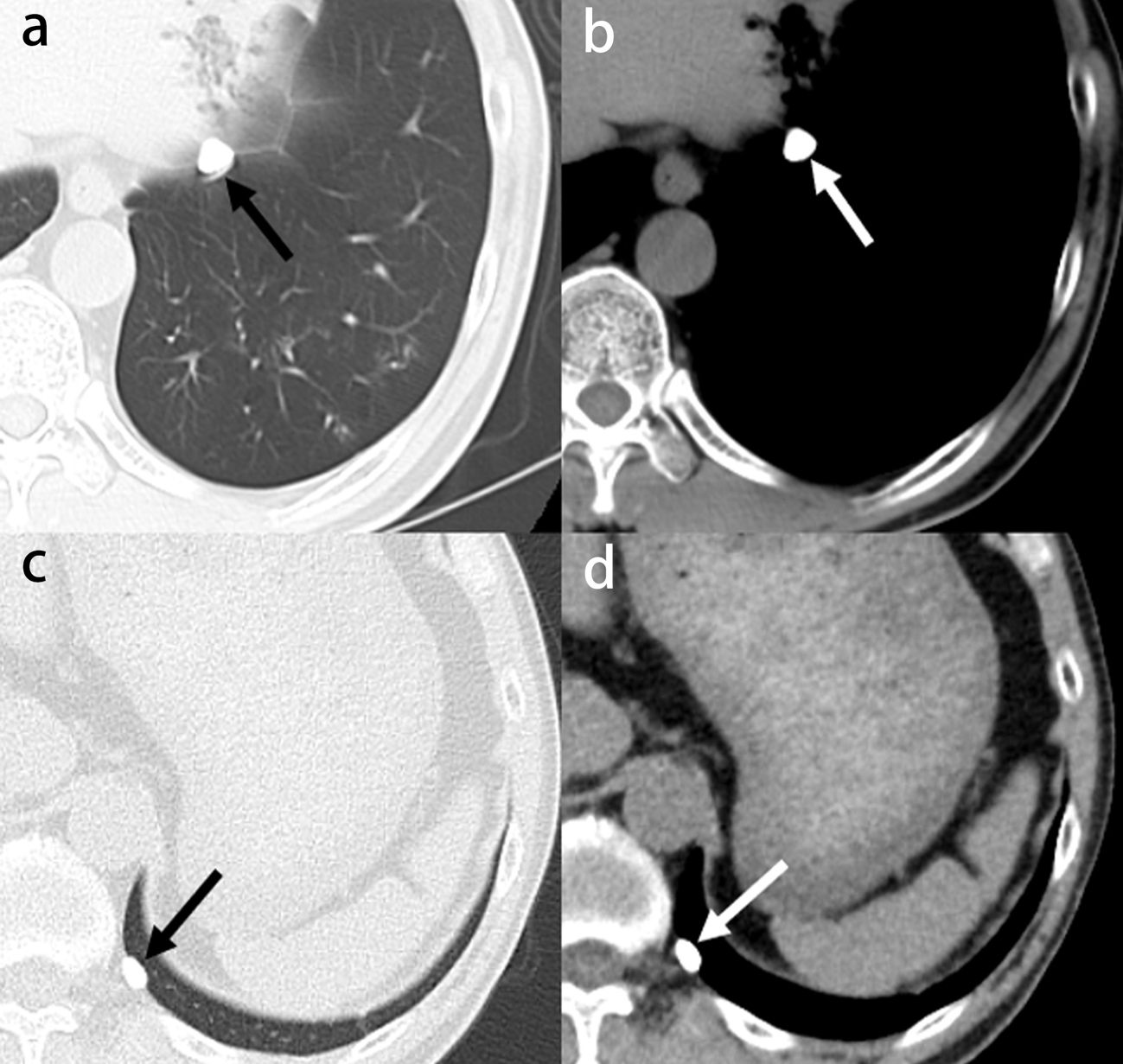


**Supplementary Fig. S13.** Thoracolithiasis in a 50-year-old man. **a, b** Initial CT images with mediastinal window setting show a smooth-marginated calcified nodule (arrow). **c, d** Follow-up CT images obtained eight years later show migration of the calcified nodule into the posteroinferior region (arrow)


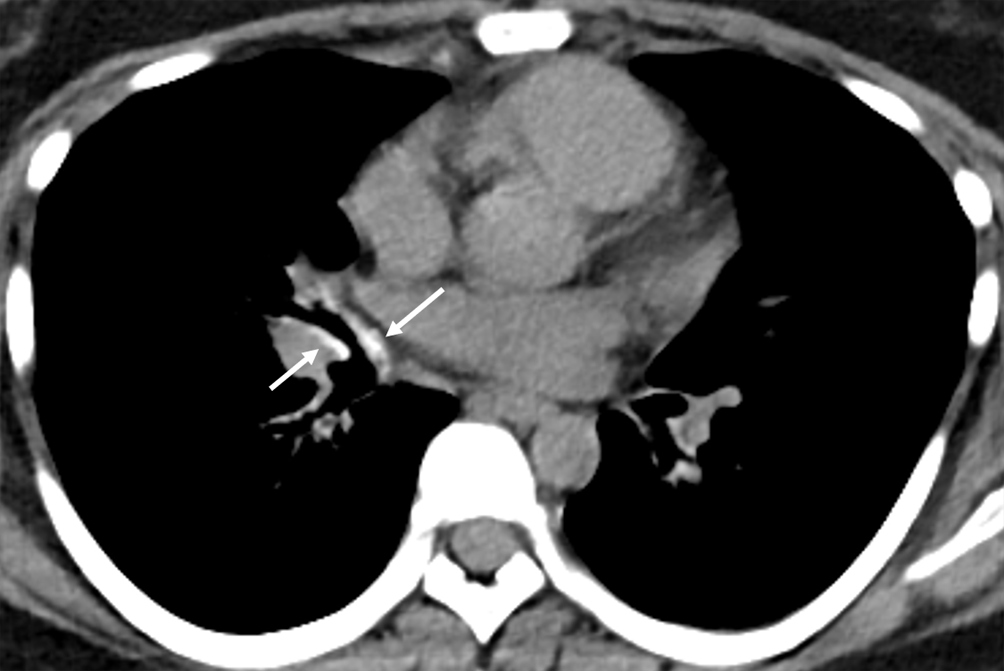


**Supplementary Fig. S14.** Tracheobronchial amyloidosis in a 22-year-old woman. CT image shows bronchial wall thickening with calcification (arrows)


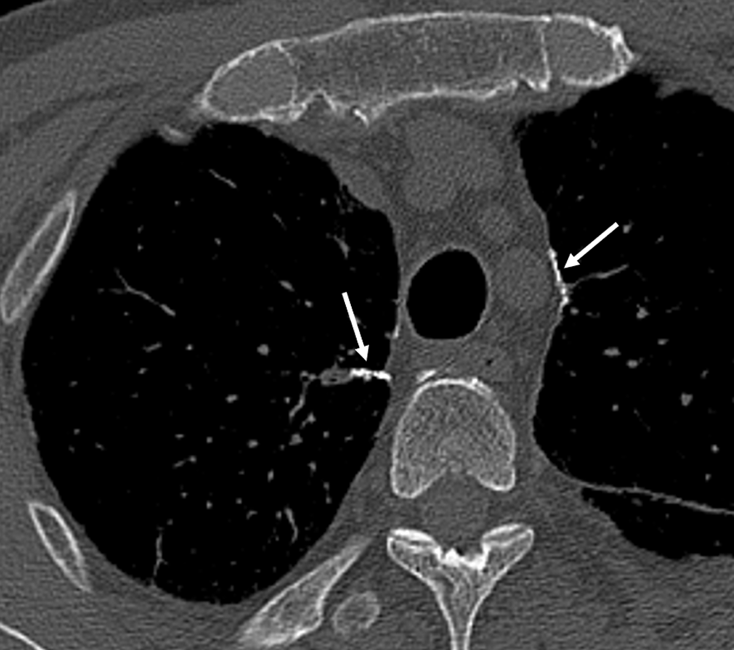


**Supplementary Fig. S15.** Postoperative status of pneumothorax in a 65-year-old man. Bone window CT image shows high-attenuation linear structures that abut the pleura, which correspond to surgical staples, are seen (arrows)


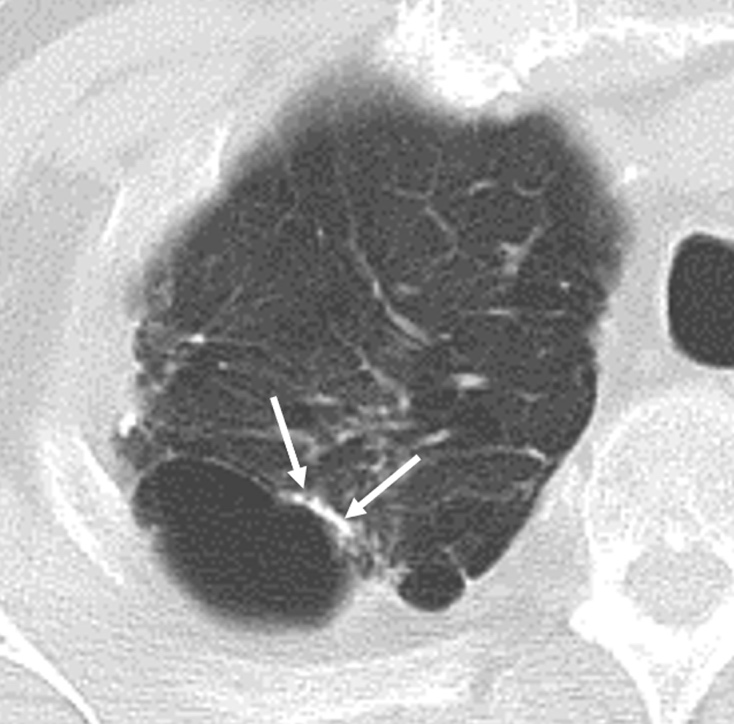


**Supplementary Fig. S16.** Inactive tuberculosis in a 70-year-old man. CT image shows linear calcification in the right upper lobe, adjacent to the bulla (arrows)


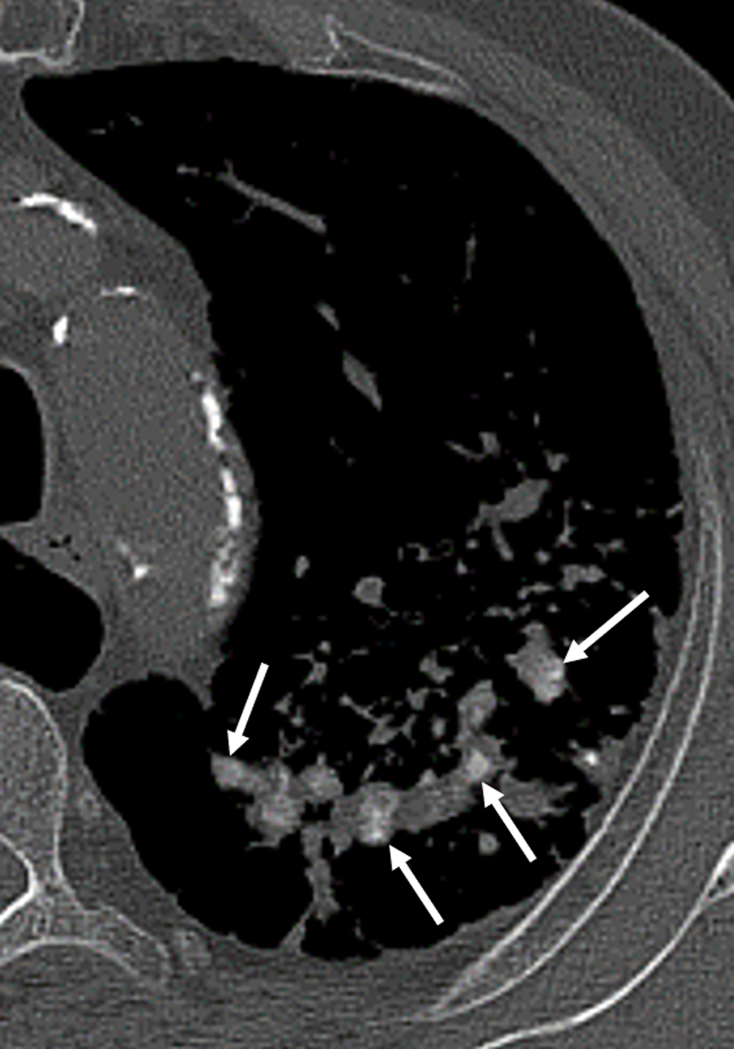


**Supplementary Fig. S17.** Inactive tuberculosis in a 90-year-old man. CT image shows numerous calcified nodules associated with soft tissues (arrows)


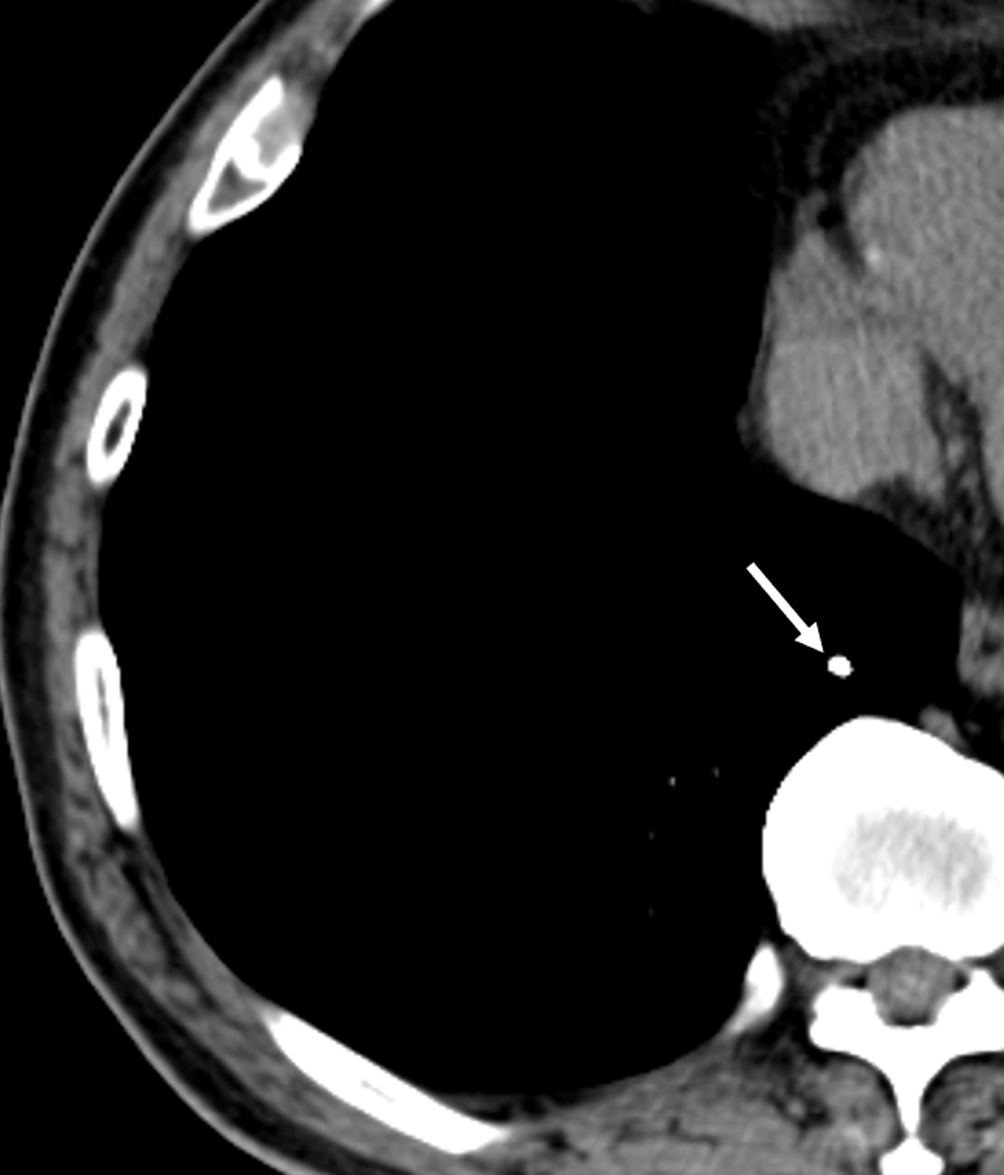


**Supplementary Fig. S18.** Ghon focus in a 60-year-old man. CT image shows nodular calcification in the periphery of the right lower lobe (arrow)


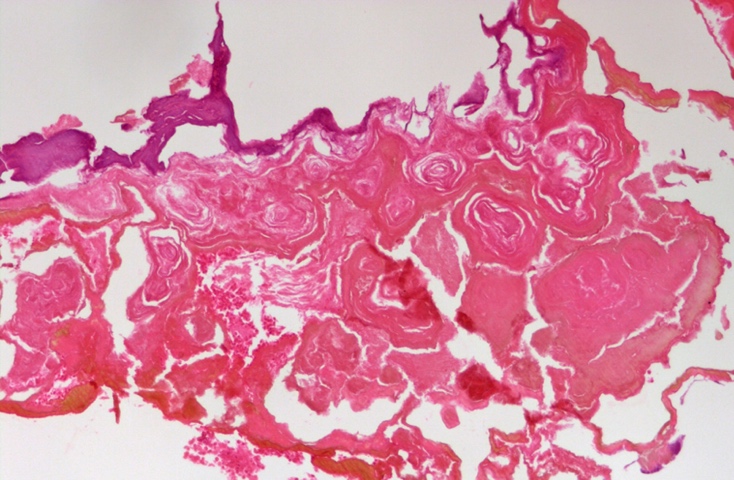


**Supplementary Fig. S19.** Broncholithiasis in a 58-year-old woman. Photomicrograph (original magnification, × 10; decalcified H-E stain) depicts a concentric decalcified stone that is considered to have originated from mucus


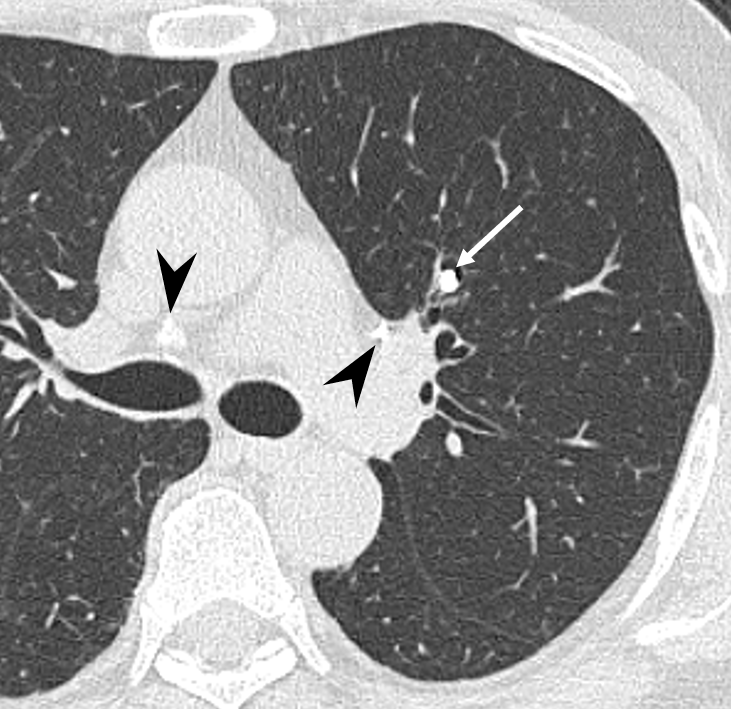


**Supplementary Fig. S20.** Broncholithiasis in a 71-year-old man. CT image shows a calcified nodule within a dilated bronchus near the left hilum (arrow). Calcified lymph nodes at the left hilum and mediastinum are also shown (arrowheads)


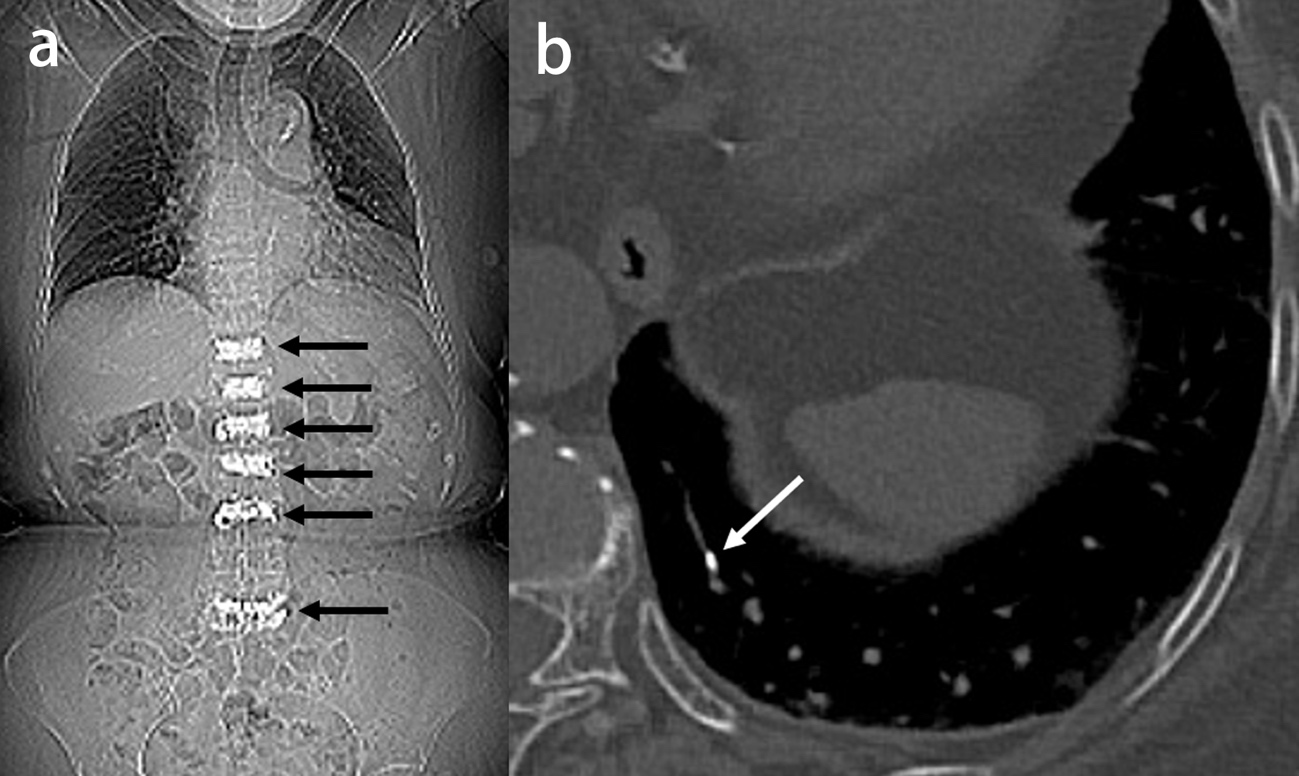


**Supplementary Fig. S21.** Cement migration due to percutaneous vertebroplasty in a 77-year-old woman. **a** CT scout view shows post-multilevel vertebroplasty (arrows). **b** Postoperative bone window CT image shows cement migration to the left lower lobe (arrow)
